# XBP1 regulates the protumoral function of tumor-associated macrophages in human colorectal cancer

**DOI:** 10.1038/s41392-021-00761-7

**Published:** 2021-10-20

**Authors:** Yahui Zhao, Weina Zhang, Miaomiao Huo, Peng Wang, Xianghe Liu, Yu Wang, Yinuo Li, Zhixiang Zhou, Ningzhi Xu, Hongxia Zhu

**Affiliations:** 1 State Key Laboratory of Molecular Oncology, National Cancer Center/National Clinical Research Center for Cancer/Cancer Hospital, Chinese Academy of Medical Sciences and Peking Union Medical College, 100021 Beijing, China; 2 Department of Colorectal Surgery, National Cancer Center/National Clinical Research Center for Cancer/Cancer Hospital, Chinese Academy of Medical Sciences and Peking Union Medical College, 100021 Beijing, China

**Keywords:** Gastrointestinal cancer, Tumour immunology

## Abstract

Macrophages are among the most abundant immune cells in colorectal cancer (CRC). Re-educating tumor-associated macrophages (TAMs) to switch from protumoral to anti-tumoral activity is an attractive treatment strategy that warrants further investigation. However, little is known about the key pathway that is activated in TAMs. In this study, infitrating CD206^+^ TAMs in CRC were sorted and subjected to RNA-seq analysis. Differentially expressed genes were found to be enriched in unfolded protein response/endoplasmic reticulum stress response processes, and XBP1 splicing/activation was specifically observed in TAMs. XBP1 activation in TAMs promoted the growth and metastasis of CRC. Ablation of XBP1 inhibited the expression of the pro-tumor cytokine signature of TAMs, including IL-6, VEGFA, and IL-4. Simultaneously, XBP1 depletion could directly inhibit the expression of SIRPα and THBS1, thereby blocking “don’t eat me” recognition signals and enhancing phagocytosis. Therapeutic XBP1 gene editing using AAV2-sgXBP1 enhanced the anti-tumor activity. Together, XBP1 activation in TAMs drives CRC progression by elevating pro-tumor cytokine expression and secretion, as well as inhibiting macrophage phagocytosis. Targeting XBP1 signaling in TAMs may be a potential strategy for CRC therapy.

## Introduction

Colorectal cancer (CRC) is a fine example of a tumor tightly associated with its immune microenvironment. Not only colitis related CRCs, but also DNA damage or mutation-derived CRCs are enhanced by inflammation in the microenvironment.^1^ The infiltration of immune cells in CRCs contributes to tumor development, progression, and therapeutic response.^2^ Immunotherapy, which targets tumor-associated immune cells, is considered the new frontier of cancer treatment. Although many approaches, including PD-1/PD-L1 targeting and CTLA-4 targeting, have been tested during clinical trials of CRC patients, these have failed to achieve satisfactory beneficial effects.^3^ Therefore, the introduction of novel immunotherapies to clinical routines may be considered a priority.

Macrophages are among the most abundant immune cells observed in CRC, and those infiltrating in the tumor microenvironment are usually defined as tumor-associated macrophages (TAMs).^4^ Infiltration of the tumor front by CD68^+^ macrophages positively correlates with improved survival in colon cancer.^5^ However, another study showed that CD68^+^ TAM density is not a significant prognostic biomarker, whereas patients with high CD206^+^ TAM density or high CD206/CD68 ratio showed significantly worse disease-free survival (DFS) and overall survival (OS) than those with a low density.^6^ Such conflicting data are attributed to the high plasticity of macrophages.^7^ Single cell RNA-seq analyses identify two distinct subsets of TAMs in the tumor microenvironment of CRC. These two subsets show different functions, one subset expresses genes involved in phagocytosis and antigen presentation, whereas the other shows a more angiogenic signature.^8^ Therefore, depleting all TAMs may be problematic. In a tumor microenvironment, macrophages may switch from an anti-tumor phenotype to a pro-tumor one depending on the environment stimuli.^9^ Thus, re-educating TAMs to switch from protumoral to anti-tumoral activity is an attractive strategy that can be used to target macrophages and warrants further investigation.^10,11^

Activated unfolded protein response (UPR) is involved in most hallmarks of cancer.^12^ The IRE1α-XBP1 pathway is the most conserved process associated with UPR. When activated, IRE1α cleaves *XBP1* mRNA, which induces the expression of activated XBP1, leading to potent transcriptional activity.^13^ Recent studies have shown that the IRE1α-XBP1 pathway is involved in immune differentiation, activation, and cytokine expression in immune cells.^14–16^ XBP1 activation has been reported in dendritic cells and T cells in ovarian cancer microenvironments, and targeting XBP1 in dendritic cells or T cells restored the anti-tumor immunity of these cells and thereby extended host survival.^17,18^ Endoplasmic reticulum (ER) stress also plays an important role in TAMs, by regulating the production of IL-6^19^ and TNF-α.^20^ IL-4-induced macrophage polarization induces ER stress, wherein the inhibition of ER stress may block polarization.^21,22^ It is reported that IRE1α-XBP1 promotes macrophage activation to M1 in white adipose tissue.^23^ The study shows that IRE1α downregulats Irf4 and Klf4 expression and suppresses M2 polarization through a mechanism that requires its RNase activity but presumably not its Xbp1 mRNA splicing activity. Another study reported that an ER stress inhibitor inhibits lipopolysaccharides (LPS)-stimulated CD206 production in macrophages.^24^ In cancers, the pro-tumor functions of TAMs promote the expression of cell surface receptors, cytokines, chemokines, and enzymes, in addition to activating Treg cells or suppressing other effector cells.^25^ Inhibition of IRE1α-XBP1 in macrophages may attenuate CD86 and PD-L1 surface expression.^26^ Therefore, targeting XBP1 pathway of TAMs may be a novel strategy for CRC immunotherapy.

The current study evaluated the role of XBP1 in TAMs associated with colon cancer. XBP1 splicing was observed in TAMs of CRC patients and mouse models. XBP1 enhanced the pro-tumor function of TAMs. Deletion of XBP1 altered the cytokine expression signature and promoted macrophage phagocytosis of tumor cells by disrupting self-recognition. Our results suggested that XBP1 in TAMs had potential as a novel therapeutic target in human colon cancer.

## Results

### TAMs infiltrating into CRC exhibit XBP1 splicing and activation

**Fig. 1:**
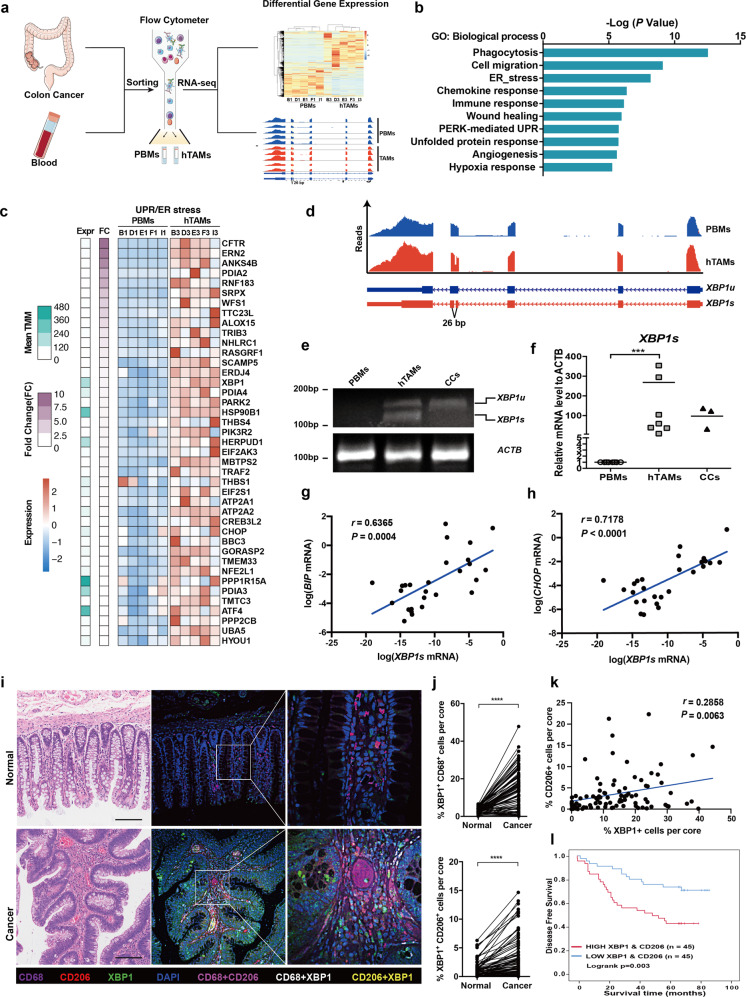
UPR/ER-XBP1 activation in human TAMs infiltrate into CRC. **a** Schematic overview of the strategy for identification of UPR/ER-XBP1 signaling pathways in hTAMs of CRC. **b** Gene Ontology (GO) term analysis of differentially regulated genes, as revealed using RNA-seq, in five paired hTAMs/PBMs samples. **c** Upregulation of genes involved in the UPR/ER stress response process. **d** XBP1 splicing in hTAMs confirmed by RNA-seq alternative mRNA splicing analysis. *XBP1u*, unspliced form; *XBP1s*, spliced form. **e** Detection of *XBP1* splicing using conventional RT-PCR and agarose gel electrophoresis. **f** Expression of *XBP1s* in PBMs, hTAMs and cancer cells evaluated by RT-qPCR. Data were normalized to endogenous levels of *ACTB*. ***P < 0.001; ANOVA test. **g**, **h** Expression of *BIP* and *CHOP* versus *XBP1s* in all hTAM samples from CRC patients (*n* = 27). **r** Spearman’s rank correlation test. **i** CD206, CD68, and XBP1 immunofluorescence stains in human CRCs and adjacent normal tissues. Scale bar: 100 μm. **j** XBP1^+^ CD68^+^ cells (upper panel), and XBP1^+^ CD206^+^ cells (lower panel) among the total number of cells in each individual core from human CRCs and adjacent normal tissues, as determined by PerkingElmer inFormTM system. ****P < 0.0001; paired *t*-test. **k** Correlation of percentage of XBP1^+^ cells with CD206^+^ in CRC patients. *r*, Spearman’s rank correlation test. **l** Kaplan–Meier surviva**l** curves for 90 CRC patients with or without high XBP1^+^CD206^+^ cells

According to the Cancer Genome Atlas (TCGA) RNA-seq data, macrophage is one of the most abundant cell types infiltrating in CRC (Supplementary Fig. S1a). TAMs accumulated in CRCs are associated with tumor progression and the efficacy of therapeutics.^5,6^ To investigate the mechanism by which TAMs interact with the tumor microenvironment, CD14^+^CD11b^+^ peripheral blood cells and CD14^+^CD11b^+^CD206^+^ intra-tumoral human CRC-associated macrophages (hTAMs) were isolated from patients with CRC and subjected to RNA-seq analysis (Fig. 1a). Although CD206^+^ macrophages accounted for the majority of CD14^+^CD11b^+^ cells isolated from the tumor, very few were observed in peripheral blood monocytes (PBMs) (Supplementary Fig. S1b, c), indicating that CD206^+^ hTAMs accumulated in the CRC tumor microenvironment. RNA-seq results showed that differentially expressed genes were enriched in ER stress and UPR process (Fig. 1b). Many known UPR/ER stress genes were upregulated in hTAMs compared with PBMs (Fig. 1c). Moreover, XBP1 splicing was detected in hTAMs from all five samples, but not in PBMs (Fig. 1d). Reverse transcriptase PCR (RT-PCR) results indicated that *XBP1* mRNA splicing in TAMs was increased compared with that in PBMs and cancer cells (Fig. 1e). Quantitative analyses consistently confirmed that the expression level of spliced *XBP1* mRNA in hTAMs was increased in hTAMs compared with that in control PBMs and cancer cells (Fig. 1f). Furthermore, expression levels of the ER stress markers, *BIP* and *CHOP*, were positively correlated with spliced *XBP1* in the hTAMs of CRC samples (Fig. 1g, h). Next, we included the multilabel immunofluorescence (MIF) of XBP1, CD206 and CD68 in CRC tissue array (Supplementary Fig. S2a and Fig. 1i). Cancerous lesions had more infiltration of XBP1^+^CD68^+^ cells (white) compared to normal tissue (14.11 ± 1.10 vs. 3.40 ± 0.18; *P* < 0.0001), XBP1^+^CD206^+^ macrophages displayed the similar pattern (3.26 ± 0.37 vs. 1.07 ± 0.15; *P* < 0.0001) (Fig. 1j). Eexpression of XBP1 and CD206 in TAMs was correlated with poor survival of CRC patients (Supplementary Fig. S2b, c). Furthermore, the infiltration of XBP1^+^ TAMs, especially XBP1^+^ CD206^+^ (representing the XBP1 activation in TAMs), in tumors was associated with shorter DFS in CRC patients, but not XBP1^+^EPCAM^+^ (representing the XBP1 activation in cancer cells) (Fig. 1k, l and Supplementary Fig. S2d, e). Moreover, the high frequency of XBP1^+^CD206^+^ TAMs (representing the XBP1 activation in TAMs) were significantly correlated with TNM stage (Fisher Exact test, *P* = 0.0314) (Supplementary Fig. S2f). XBP1 expression was highly correlated with the TAM markers, CD206 and CD163, which was also confirmed using a public GEO databases (GSE14333) (Supplementary Fig. S2i). Expression of XBP1 and CD206 was strongly associated with shorter DFS in GEO databases GSE38832 (Supplementary Fig. S2g, h) and GSE14333 (Supplementary Fig. S2j, k).

**Fig. 2:**
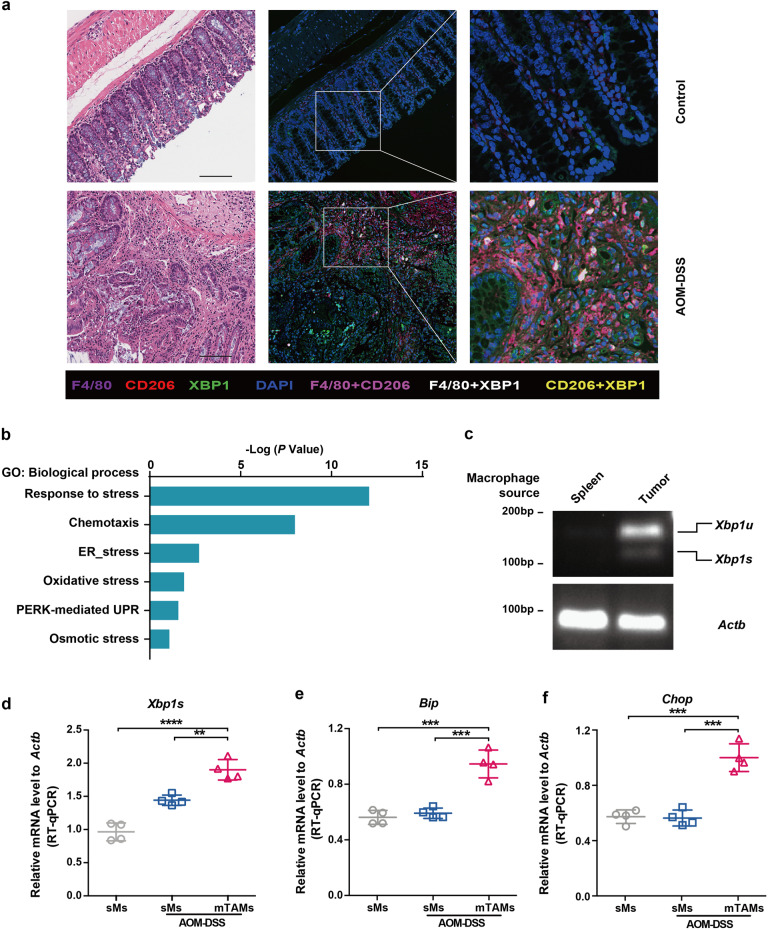
XBP1 activation in AOM-DSS-induced colorectal cancer-associated macrophages. **a** F4/80, CD206, and XBP1 immunofluorescence in colon sections from healthy and AOM-DSS mice. Scale bar: 100 μm. **b** Gene Ontology (GO) term analysis of differentially regulated genes, as revealed using RNA-seq, in four paired mTAMs and spleen macrophages (sMs). **c** *Xbp1* splicing was evaluated using conventional RT-PCR and agarose gel electrophoresis. **d** Expression of *XBP1s* in spleen macrophages (sMs) and mTAMs evaluated via RT-qPCR. Data were normalized to endogenous levels of *ACTB*. **e**, **f** Expression of the UPR/ER stress response transcripts *Bip* and *Chop* determined by RT-qPCR. Data normalized to endogenous levels of *ACTB*. (*n* = 4 mice per group); **P < 0.01, ***P < 0.001, ****P < 0.0001; ANOVA test

Furthermore, an AOM-DSS-induced CAC model was established (Supplementary Fig. S3a). Following three cycles of induction, tumors became visible in the colon, upon which the mice were sacrified (Supplementary Fig. S3b, c). Immunofluorescence staining demonstrated that XBP1 was expressed in macrophages infiltrating in cancerous lesions (XBP1^+^F4/80^+^ and XBP1^+^CD206^+^ cells) but not in F4/80^+^ macrophages infiltrating in normal tissues (Fig. 2a). Mouse TAMs (mTAMs) from tumor colorectal lesions and spleen monocytes were sorted (Supplementary Fig. S3d) and applied to RNA-seq analysis. Differentially expressed genes were enriched in stress-related processes (Fig. 2b). RT-PCR and agarose gel electrophoresis indicated that although mTAMs sorted from tumors of mouse AOM-DSS model contained spliced *Xbp1*, no splicing was detected in macrophages sorted from the spleen (Fig. 2c). *Xbp1s* expression was significantly higher in mTAMs than that in control spleen monocytes (Fig. 2d). ER stress markers, *Bip* and *Chop*, were also upregulated in mTAMs (Fig. 2e, f). The above results indicated that although macrophages derived from tumor tissues showed XBP1 activation, those derived from normal tissues did not. Taken together, TAMs infiltrating in CRC exhibit XBP1 splicing and activation.

### XBP1 activation in TAMs promotes the growth and metastasis of CRC

**Fig. 3:**
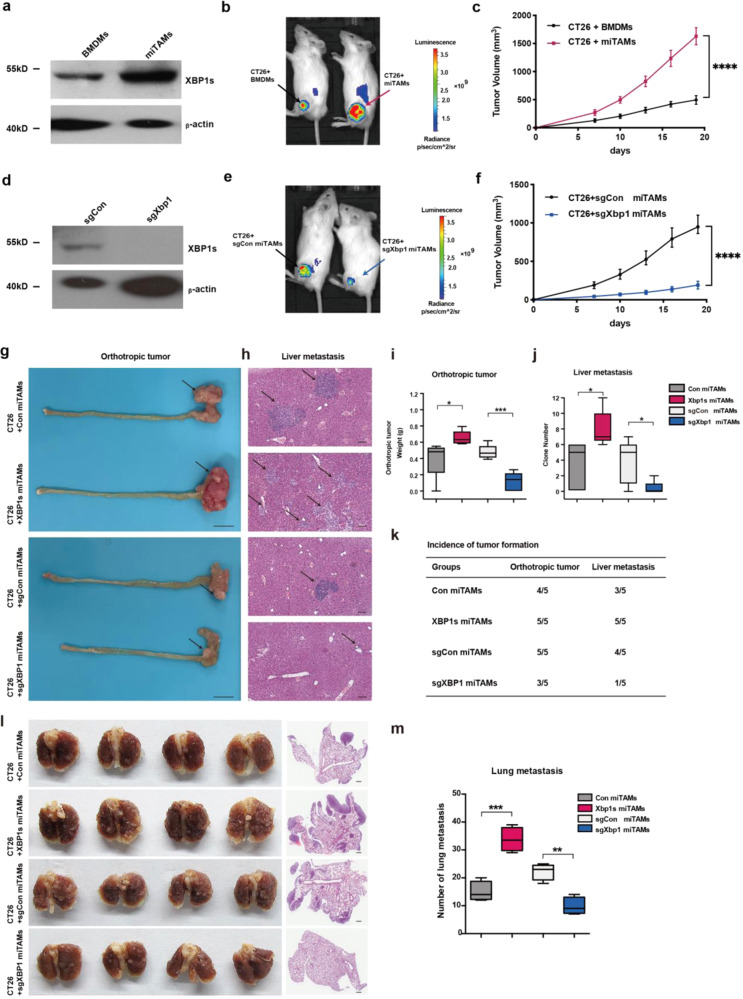
Effect of XBP1 activation on the pro-tumor function of TAMs. **a** Western blot analysis of XBP1s expression in indicated macrophages. **b** Representative micrograph showing tumor formation in NOD/SCID mice injected subcutaneously (s.c.) with luciferase tumor cells (CT26-luciferase) and two groups of macrophages: CT26 + BMDMs (black arrows) and CT26 + miTAMs (magenta arrows). **c** Growth curves of the two groups in **b**. **d** Western blot analysis of XBP1 expression in sgCon or sgXbp1 TAMs. **e** Representative photograph showing tumor formation in NOD/SCID mice injected s.c. with luciferase tumor cells (CT26-luciferase) and two groups of miTAMs: CT26 + sgCon miTAMs (black arrows); and CT26 + sgXbp1 miTAMs (blue arrows). **f** Growth curves of the two groups in **e**. ****P < 0.0001; Repeated measurement and analysis. **g** CT26 cells, mixed with: Con TAMs; Xbp1s TAMs; sgCon TAMs; and sgXbp1 TAMs, were orthotopically injected into the wall of the cecum (*n* = 5 mice per group). Macroscopic appearance of the CRC orthotopic tumors with each indicated treatment. Black arrows indicate macroscopic polyps. Scale bar, 1 cm. **h** Representative HE staining of liver metastasis in mice xenografted of the four groups in **g**. Scale bar, 100 μm. **i**, **j** Statistical analysis of the orthotopic CRC tumor weight (**i**) and the clone number of liver metastasis (**j**). *P < 0.05, ***P < 0.001; *t*-test. **k** Incidence of orthotopic CRC tumor formation and liver metastasis analysis. **l** Representative images of CT26 pulmonary metastases induced by tail vein injection in NOD/SCID. CT26 cells were mixed with: Con miTAMs; Xbp1s miTAMs; sgCon miTAMs; and sgXbp1 miTAMs. HE staining demonstrating the histology of tumors formed in the lungs; scale bar, 500 μm. **m** Pulmonary metastatic nodule numbers in **i**. (*n* = 4 mice per group); **P < 0.01, ***P < 0.001; *t*-test

We next investigated the contribution of XBP1 in TAMs associated with tumor growth and metastasis. TAMs enhanced tumor growth of the subcutaneously injected luciferase tumor cells (CT26-luciferase) in NOD/SCID mice (Fig. 3a–c), while knockout of XBP1 in miTAMs inhibited tumor growth (Fig. 3d–f and Supplementary Fig. S4a–c). Inhibition of XBP1 splicing by IRE1α inhibitor 4μ8c also diminished tumor-promoting function of TAMs (Ana-1 conditioned medium [CM]) (Supplementary Fig. S4d–f). Next, we determined whether XBP1 activation in TAMs promoted metastasis of CRC cells. Metastasis of CT26 cells was analyzed using the Boyden chamber assay (Supplementary Fig. S4g). While TAMs (Ana-1 CM) enhanced the migration of CT-26 cells, knocking out of XBP1 in TAMs inhibited CT26 cell migration (Supplementary Fig. S4h, i). NOD/SCID mice were pretreated with clodronate liposomes for two weeks to eliminate intrinsic macrophages. CT26 cells mixed with Con TAMs, Xbp1s TAMs, and sgCon TAMs, and sgXbp1 TAMs, were injected orthotopically into the wall of the cecum. After four weeks, incidence of orthotopic CRC tumor formation and HE staining of liver metastasis analysis were observed (Fig. 3g, h). Compared with control group, mice injected with miTAMs XBP1s-overexpressing miTAMs showed an increase in orthotopic tumor formation (4/5 vs. 5/5) and liver metastasis (3/5 vs. 5/5). Conversely, mice harboring TAMs lacking XBP1 showed reduced orthotopic tumor formation (5/5 vs. 3/5) and liver metastasis (4/5 vs. 1/5) compared with control mice. Quantification of the orthotopic CRC tumor weight and the clone number for liver metastasis confirmed that co-injection with XBP1s-overexpression TAMs could lead to a significant increase in the tumor burden of CRC orthotopic tumor burden and liver metastasis, and vice versa (Fig. 3g–k). Furthermore, spleen injection model of liver metastasis also indicated that mice injected with XBP1s-overexpressing miTAMs showed an increase in liver metastasis, compared with control mice (25.92 ± 2.41 vs. 9.76 ± 1.85; *P* < 0.01), whereas mice harboring TAMs lacking XBP1 showed reduced liver metastasis compared with control mice (1.13 ± 0.51 vs 11.47 ± 1.13; *P* < 0.001); (Supplementary Fig. S4j, k). Similarly, CT-26 injected via the tail vein promoted the formation of metastatic nodules in the lung. Histological staining confirmed that the cells were cancerous (Fig. 3l). Mice injected with XBP1s-overexpressing miTAMs displayed increased number of lung nodules compared with those in control mice (33.75 ± 2.14 vs 15.00 ± 1.78; *P* < 0.001). In contrast, mice lacking XBP1 in miTAMs showed reduced number of lung nodules compared with control mice (9.75 ± 1.55 vs 22.25 ± 1.49; *P* < 0.01); (Fig. 3m). Taken together, XBP1 activation in TAMs was necessary for the growth and metastasis of CRC in mouse models.

### XBP1 activation regulates the cytokine expression signature of TAMs

**Fig. 4:**
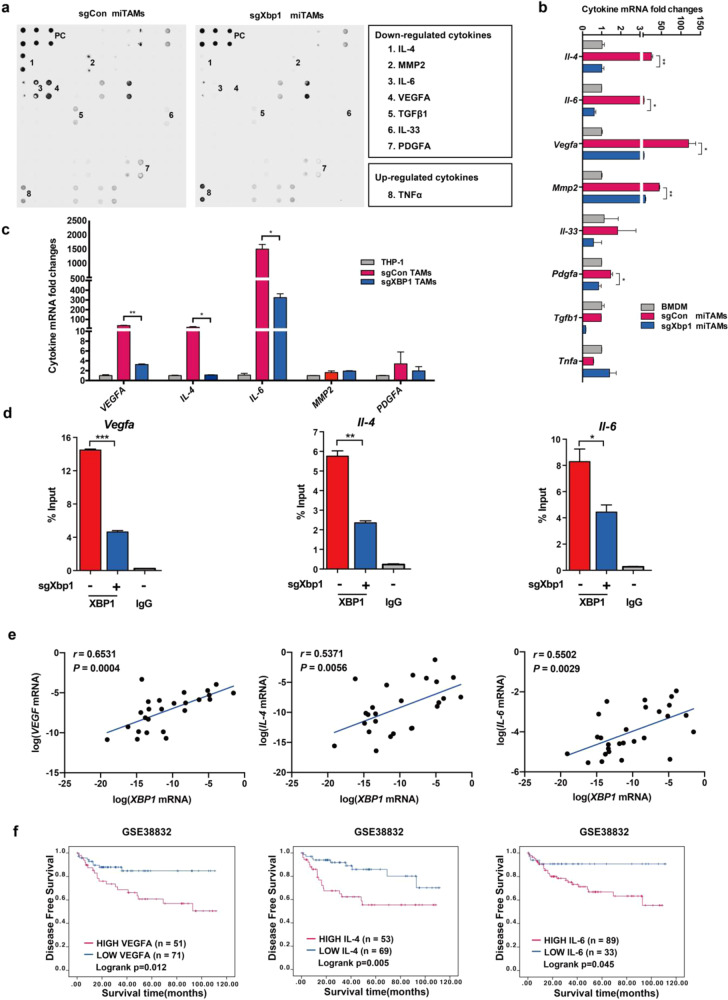
Cytokines production induced by XBP1 activation in TAMs. **a** Representative cytokine arrays for sgCon miTAMs and paired sgXbp1 miTAMs supernatants. **b** Relative mRNA levels of cytokines in BMDMs, sgCon miTAMs, and paired sgXbp1 miTAMs validated by RT-qPCR. **c** Expression of cytokines in human TAMs. Human macrophages from THP-1 cells were induced to TAMs via incubation in condition medium of HCT116 cells, and relative mRNA levels of cytokines in THP-1, sgCon or sgXBP1 TAMs were validated by RT-qPCR. **d** ChIP-qPCR experiments measuring XBP1 binding on *Vegfa*, *Il-4*, and *Il-6* segments. Bars represent mean ± SD of three experimental replicates. *P < 0.05, **P < 0.01, ***P < 0.001; *t*-test. **e** Expression of VEGFA, IL-4, and IL-6 versus XBP1s in all hTAM samples from CRC patients (*n* = 27). *r*, Spearman’s rank correlation test. **f** Kaplan–Meier survival curves **f**or CRC patients with or without high expression levels of VEGFA, IL-4 and IL-6 in the GEO online database (GSE38832). The optimal survival cut point was determined via X-Tile statistical software

Cytokines released by macrophages influence cancer cell proliferation and migration.^27,28^ To determine whether XBP1 promotes tumor growth by regulating cytokine expression in TAMs, cell supernatants of TAMs and XBP1-knockout TAMs were applied to cytokine array assay. The concentrations of IL-4, IL-6, and VEGFA, decreased significantly in XBP1 knocked-out miTAMs, whereas TNFα increased (Fig. 4a). RT-qPCR indicated that expression of *Il-4*, *Il-6*, *Mmp2*, *Vegfa*, *Il-33*, *Pdgfa*, and *Tgfb1* was upregulated in miTAMs compared with that in original bone marrow-derived macrophages (BMDMs), but decreased when XBP1 was knocked out. In contrast, *Tnfa* expression, which was decreased in TAMs, was found to be increased in sgXBP1 miTAMs (Fig. 4b). RT-qPCR showed similar results in tumor condition medium induced human macrophage THP-1 cells (Fig. 4c). ELISA results indicated that the concentrations of VEGFA and IL-6 were upregulated in TAMs (THP-1 cell induced by HCT116 CM; THP-1 CM) compared with those in THP-1 cells, but downregulated upon XBP1 knockout (Supplementary Fig. S5a, b). Meanwhile, track view of ChIP-seq density profiles in the GSE86048 database indicated that XBP1 knockdown reduced the binding efficacy of *Vegfa*, *Il-4* and *Il-6* sequences (Supplementary Fig. S5c). Nuclear extracts from control TAMs or sgXbp1 TAMs were subjected to ChIP using anti-XBP1 antibodies. The promoter regions of *Vegfa*, *Il-4* and *Il-6* were enriched in the immunoprecipitates with anti-XBP1 antibody, but knock-down of XBP1 decreased the binding of these promoters (Fig. 4d). Moreover, RT-qPCR showed that the expression levels of *VEGFA*, *IL-4* and *IL-6* were positively correlated with that of *XBP1* in hTAMs (M2-like) derived from CRC patients (Fig. 4e). A similar relationship was also found in the tumor samples of CRC patients (GSE38832), as shown in Supplementary Fig S5d. High expression of VEGFA, IL-4 and IL-6 was associated with shorter DFS in CRC patients (GSE38832) (Fig. 4f). These results demonstrate that XBP1 directly regulates the transcription signature of cytokines in macrophages and thereby enhances cancer progression.

### XBP1 inhibition promotes macrophage phagocytosis by disturbing the self-recognition

**Fig. 5:**
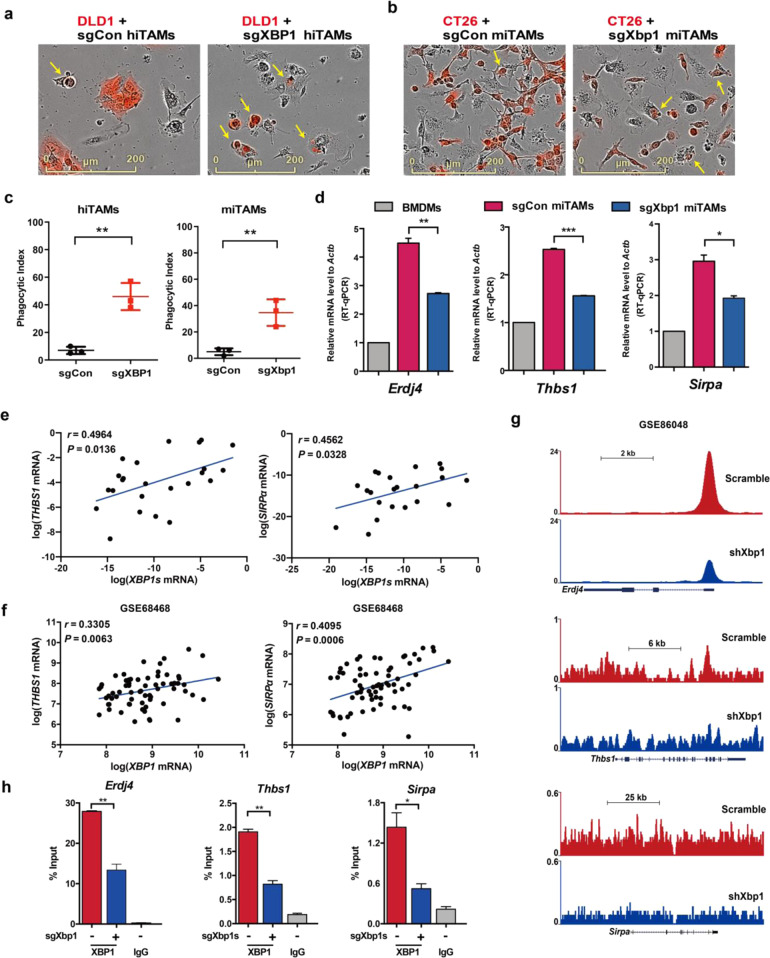
Effect of XBP1 on macrophages phagocytosis. **a** Representative images of phagocytosis assays using RFP-labeled human CRC cell, DLD1cells (DLD1-RFP) and sgCon or sgXBP1 hiTAMs (*n* = 3). Yellow arrows denote phagocytic events. Scale bar = 200 μm. **b** Representative images of phagocytosis assays using RFP-labeled mouse CT26 cells (CT26-RFP) and sgCon or sgXbp1 miTAMs (*n* = 3). Yellow arrows denote phagocytic events. Scale bar = 200 μm. **c** Results of phagocytosis assays of the two groups in **a**, **b**. **P < 0.01; *t*-test. **d** Relative mRNA levels of *XBP1* and phagocytosis-associated genes in BMDM, sgCon miTAMs and sgXbp1 miTAMs, validated by RT-qPCR. Bars represent mean ± SD of three experimental replicates. *P < 0.05, **P < 0.01, ***P < 0.001; *t*-test. **e** Expression of *THBS1* and *SIRPα* versus *XBP1* in TAMs sorted from CRC patients (*n* = 27 total). *r*, Spearman’s rank correlation test. **f** Correlation of *XBP1* with *THBS1* and *SIRPα* in CRC patients. The association was analyzed using coefficient measures of the linear relationships in the public GEO database (GSE68468). **g** Track view of *Erdj4*, *Thbs1*, and *Sipra* ChIP-seq density upon silencing of Xbp1 in the ChIP-seq online database (GSE86048). **h** ChIP-qPCR experiments measuring XBP1 binding on *Erdj4*, *Thbs1*, and *Sipra* segments. Bars represent mean ± SD of three experimental replicates. *P < 0.05, **P < 0.01. *P*-values were determined using *t*-test

In addition to releasing cytokines that modulate tumor progression, TAMs clear tumor cells directly via phagocytosis. To evaluate whether XBP1 affects phagocytosis, we co-cultured RFP-labeled DLD1 with hiTAMs (Peripheral blood mononuclear cell (PBMC)-derived macrophages), and cocultured RFP-labeled CT26 with miTAMs (BMDMs). Following 8 h of co-culture, compared with the sgCon TAMs, the RFP-labeled cancer cells (red) were efficiently phagocytosed by sgXBP1 hiTAMs or sgXbp1 miTAMs, indicating that XBP1 knockout increased the phagocytic capacity of TAMs remarkably (Fig. 5a–c). The phagocytic activity of macrophages is regulated by activating (“eat”) as well as inhibitory (“do not eat”) signals. CD47, a widely expressed transmembrane glycoprotein, suppresses phagocytosis by binding to SIRPα and THBS1 on the surface of macrophages.^29,30^ SIRPα and THBS1 levels were upregulated in miTAMs compared with those in BMDMs, but inhibition of XBP1 significantly reduced the expression levels (Fig. 5d). Similar results were observed in THP-1 cells (Supplementary Fig. S6a). The expression pattern of SIRPα and THBS1 were similar to that of ERDJ4, a target of XBP1. RT-qPCR showed that the expression levels of *SIRPα* and *THBS1* were positively correlated with that of *XBP1* in hTAMs derived from CRC patients (Fig. 5e). A similar relationship was also found in the tumor samples of CRC patients (GSE68468, GSE38832), as shown in Fig. 5f and Supplementary Fig. S6c. THBS1 and SIRPα expression was correlated with poor prognoses for CRC patients (Supplementary Fig. S6d). Then, we sought to investigate whether SIRPα and THBS1 were transcriptional targets of XBP1. Track view of ChIP-seq density profile indicated that *Erdj4*, *Thbs1*, and *Sirpa* (GSE86048) in the livers of XBP1-knockdown mice showed reduced binding efficacy (Fig. 5g). ChIP experiments showed that the promoter regions of *Erdj4*, *Thbs1*, and *Sirpa* were enriched in the immunoprecipitates with anti-XBP1 antibody but knockout of Xbp1 led to decreased binding of these promoters in miTAMs (Fig. 5h). Therefore, XBP1 directly regulate the transcription of THBS1 and SIRPα. XBP1 inhibition promoted macrophage phagocytosis of tumor cells by disturbing the self-recognition of TAMs.

### Therapeutic knockout of XBP1 enhances anti-tumor activity of TAMs

**Fig. 6:**
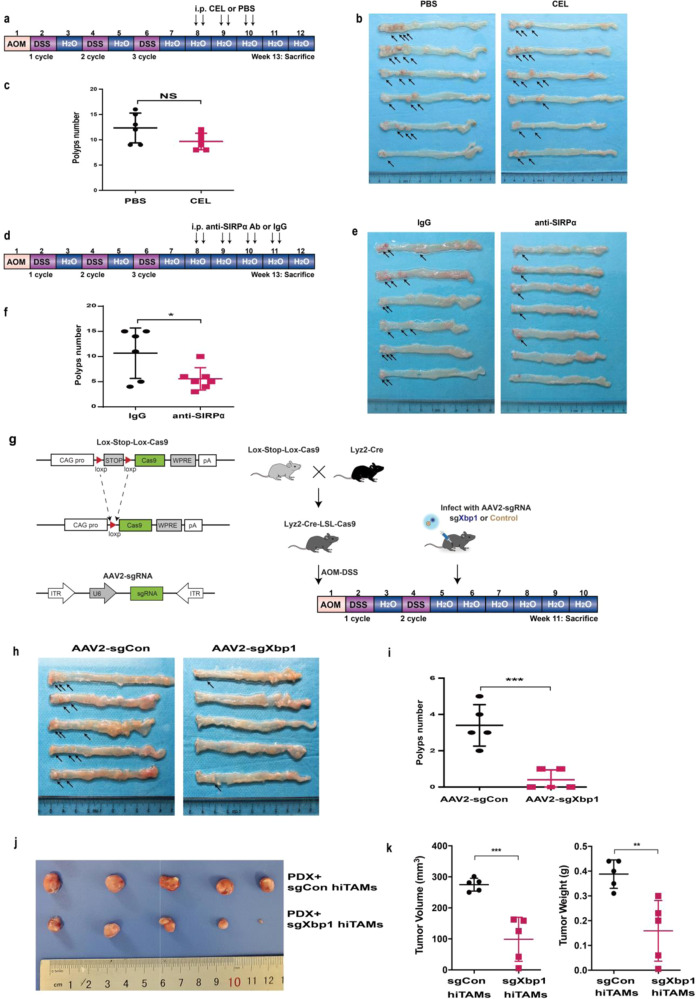
Therapeutic effects of targeting UPR/ER-XBP1 signaling in TAMs. **a** Schematic overview of macrophage depletion in an AOM-DSS model. **b** Pictures of the whole colons. The arrowhead indicates macroscopic polyps. **c** Mean macroscopic polyp number in whole colons. **d** Schematic overview of the administering of anti-SIRPα antibodies during late stages of the AOM-DSS model. Mice were treated with anti-SIRPα antibodies (8 mg/kg) vs. control IgG twice/week after the third DSS cycle for 4 weeks. Colons were removed at week 13 following AOM injection. **e** Pictures of the whole colon. The arrowhead indicates macroscopic polyps. **f** Mean macroscopic polyp numbers in whole colons (*n* = 6 for IgG and *n* = 7 for anti-SIRPα Ab). **g** Schematic representing the generation of mice with genetically targeted and deficient XBP1 in TAMs. Mice were treated with AAV2-sgXbp1 (5 × 10^11^/mouse, i.p.) vs. control AAV2-sgCon twice/week after the second DSS cycle for 4 weeks. Colons were removed at week 11 after AOM injection. **h** Pictures of the whole colon. The arrowhead indicates macroscopic polyps. **i** Mean macroscopic polyp number in whole colons (*n* = 5 for sgCon and *n* = 5 for sgXbp1). NS = *P* > 0.05, *P < 0.05, ***P < 0.001. *P*-values were determined using *t*-test. **j** Representative photograph showing tumor formation in NOD/SCID mice injected s.c. with CRC PDX and two groups of TAMs: PDX + sgCon hiTAMs; and PDX + sgXBP1 hiTAMs. **k** Tumor volumes and tumor weights were resected and measured 3 weeks later. **P < 0.01, ***P < 0.001; Mann–Whitney *U*-test

Then, the role of XBP1 in TAMs in tumor progression was evaluated via AOM-DSS model. Clodronate liposome (clodrolip) reduces the number of macrophages infiltrating tumors, limits tumor metastasis in lung cancer.^31^ Therefore, we depleted all macrophages using clodrolip injections in AOM-DSS mouse model (Fig. 6a), but there was no significant difference between the polyp numbers of treatment and control groups (Fig. 6b, c). Phagocytosis checkpoints are now considered as promising targets for cancer immunotherapy.^32^ CD47/SIRPα axis is one of the most important pathways, which enables tumor cells to evade phagocytosis by macrophages. Blockage of the CD47/SIRPα stimulates phagocytosis of cancer cells by macrophages in vitro and inhibits tumor growth in vivo.^33^ Therefore, we used therapeutic SIRPα antibodies to treat AOM-DSS-induced CRC. Tumor numbers were significantly reduced by SIRPα antibody treatment (Fig. 6d–f). Previous results showed that targeting XBP1 inhibited the growth and metastasis of CRC in mouse models. We evaluated the therapeutic effect of XBP1 silencing on AOM-DSS model. To selectively target XBP1 in macrophages, sgRNA-Xbp1 adeno-associated virus was produced as described in methods. Lyz2-Cre-LSL-Cas9 mice were inoculated with AAV2- sgXbp1 one week following the second DSS cycle, and euthanized five weeks later (Fig. 6g). To test the efficacy and specificity of AAV2-sgXbp1 on the expression of XBP1 in mTAMs, we determined the MIF of F4/80, CD206, and XBP1 in colon sections from AAV2-sgCon and AAV2-sgXbp1 AOM-DSS mice. We detected the expression of XBP1 in colorectal cancer tissue from mice model treateds with AAV2-sgXbp1, as shown in Supplementary Fig. S7a–d; the expression of XBP1 decreased in F4/80^+^ macrophages but not in epithelium cells. The result indicated that the anti-tumor function was a result of knocking out XBP1 from TAMs but not from tumor cells. Conditional deletion of XBP1 in TAMs significantly inhibited tumor formation in AOM-DSS mouse model (Fig. 6h, i). To test the therapeutic effects of targeting XBP1 in macrophages in human models, we employed CRC PDXs to for co-injection with hiTAMs (Fig. 6j, k). Knockout of XBP1 in hiTAMs inhibited PDX tumor growth. Further, mTAMs isolated from mice treated with AAV2-sgXbp1 showed reduced expression and secretion of IL-4, IL-6 and VEGFA (Supplementary Fig. S7e, f). The expression of recognition signals (*Sipra*, *Thbs1*, *Pd-l1*, *Pd-1*, *Siglec-10*, and *H2-k1*), especially *Sipra*, also decreased in AAV2-sgXbp1 mTAMs (Supplementary Fig. S7g). These data indicated that abrogating XBP1 function in TAMs could reduce the expression of tumor-promoting cytokines and also inhibit the “don’t eat me” signals of macrophages in vivo. Together, these data indicated that targeting XBP1 in TAMs inhibit the progression of CRC.

## Discussion

We developed a potential strategy for CRC immunotherapy via the modulation of XBP1 activation in TAMs associated with CRC. XBP1 depletion in TAMs changed the cytokine expression patterns and disrupted the self-recognition capacity of TAMs, thereby enhancing the anti-tumor activity of macrophages, which led to the inhibition of tumor progression.

UPR activation endows malignant cells with greater tumorigenic, metastatic, and drug resistant capacity.^17^ XBP1 is overexpressed in colon cancer cells, whereas it was found to be unreactive in normal colon epithelial cells.^34^ In tumor microenvironment, nutrient deprivation, oxygen limitation, high metabolic demand, and oxidative stress, disturb the protein-folding capacity of the ER, thereby provoking a cellular state of ER stress.^17,35,36^ The IRE1α-XBP1 pathway may reasonably be considered a target candidate for cancer treatment. However, targeting ER stress in cancer cells has not yielded satisfactory results. Inhibiting IRE1 RNase activity in patient-derived xenograft models, using small molecule inhibitor 8866, restrained MYC-overexpressing tumor growth but did not reduce tumors expressing MYC.^37^ In APC^min/+^ mouse model, epithelial-specific XBP1 deficiency was associated with a profound increase in tumorigenesis in CAC.^38^ The current study determined that XBP1 was present in both cancer cells and hTAMs sorted from CRC samples, whereas the amount of XBP1 present in TAMs was significantly higher than that present in cancer cells. Knockout of XBP1 in TAMs significantly inhibited the progression and metastasis of colon cancer. Thus, XBP1 activation in TAMs, but not in cancer cells, might be a potential immunotherapeutic target for CRC.

Shan et al. reported that IRE1α promotes macrophage polarization to M1 in white adipose tissue. They showed that IRE1α downregulates Irf4 and Klf4 expression and suppresses M2 polarization, through a mechanism that requires its RNase activity, but presumably not its Xbp1 mRNA splicing activity.^23^ However, our results showed that XBP1 could upregulate the pro-tumorigenic cytokines expression and reduce the phagocytosis, which could enhance the pro-tumorigenic function of macrophages (M2-like TAMs) in the tumor microenvironment. Therefore, whether ER stress promotes macrophage polarization to the M1 or M2 phenotype depends on the exact inflammation or tumor microenvironment stimulation in which the macrophage are located at that time.

The pro-tumor function of TAMs is executed by expressing cell surface receptors, cytokines, chemokines, and enzymes that activate Treg cells or suppress other effector cells.^25^ Therefore, we investigated whether XBP1 signaling contributes to the expression signature of TAMs. Inhibition of XBP1 in TAMs downregulated the expression of pro-tumor cytokines, such as IL-4, IL-6, MMP2 and VEGFA. This converted TAMs from a pro-tumor function to an anti-tumor function. As a transcriptional factor, XBP1 regulates the expression of cytokines in response to stimuli,^20^ binds directly to the promoter regions of IL-6 and VEGFA and activates its expression,^39,40^ and this observation was also confirmed by our study. Bevacizumab (therapeutic anti-VEGF antibodies) is used, in combination with chemotherapy or targeting therapy, to clinically treat colon cancer.^41^ IL-6 is the key component of the cytokine release syndrome in immunotherapy-induced adverse events, and combination of IL-6 antibody (or IL-6R antibody) is now widely used in CAR-T cell therapy.^42^ Therefore, these results may provide a theoretical basis suggesting that targeting XBP1 alone might be an alternative strategy to targeting a combination of IL-6 and VEGFA for CRC therapy.

Besides secreting soluble cytokines, macrophages participate in eliminating tumors via phagocytosis.^43^ Macrophage-based phagocytosis relies on the recognition of “eat me” or “don’t eat me” signals emanating from target cells. Cancer cell surfaces express “don’t eat me” signals and interact with recognition molecules on the surface of macrophages. Such interactions result in the blockage of phagocytosis. These signals include CD47/SIRPα,^29^ PD-L1/PD-1,^44^ CD24/Siglec-10,^45^ and MHC-I/LILRB1.^46^ We found that XBP1 overexpression dramatically decreased phagocytosis by TAMs, but XBP1 knockout increased the phagocytic capacity of TAMs. Moreover, inhibition of XBP1 significantly reduced the expression of SIRPα and THBS1 in TAMs. ChIP results indicated that XBP1 directly regulates the transcription of SIRPα and THBS1. Our study reveales a new mechanism of XBP1 in TAMs that promotes phagocytosis of tumor cells by disrupting self-recognition.

CRC exhibits an immunosuppressive microenvironment similar to the other cancers. Immunotherapies that utilize PD-1/PD-L1 antibodies and CTLA-4 are currently undergoing clinical trials with some encouraging results.^47^ But the benefits seen in CRC have been limited to a few groups of patients with MSI-H tumors, which makes up only 3.5-17% of all CRCs.^48,49^ Therefore, a new therapeutic approach that does not only benefit a select group of CRC patients is highly desired. First, we treated CRC in AOM-DSS mouse model with clodrolip and found that the effect was not significant. This could be due to the depletion of all macrophages, including both the “M1” and “M2” macrophages by clodrolip. Thus, selectively diminishing the tumor-promoting function of TAMs might be a better strategy. Targeting TAM inhibitory (“do not eat”) signals-CD47/SIRPα axis by therapeutic SIRPα antibodies could inhibit tumor formation inAOM-DSS-induced CRC. However, our results showed that targeting XBP1 in TAMs were more effective due to its dual function of inhibiting tumor-promoting cytokines and recognition signals. Furthermore, targeting XBP1 in TAMs inhibited the expression of PD-L1,^50^ SIRPα, and THBS1, together with the blockage of cytokines, simulated the effects of a powerful combination of several popular therapeutic programs. AAV2-sgXbp1, which conditionally deletes XBP1 in TAMs, dramatically inhibited tumor formation in AOM-DSS mouse model.

**Fig. 7:**
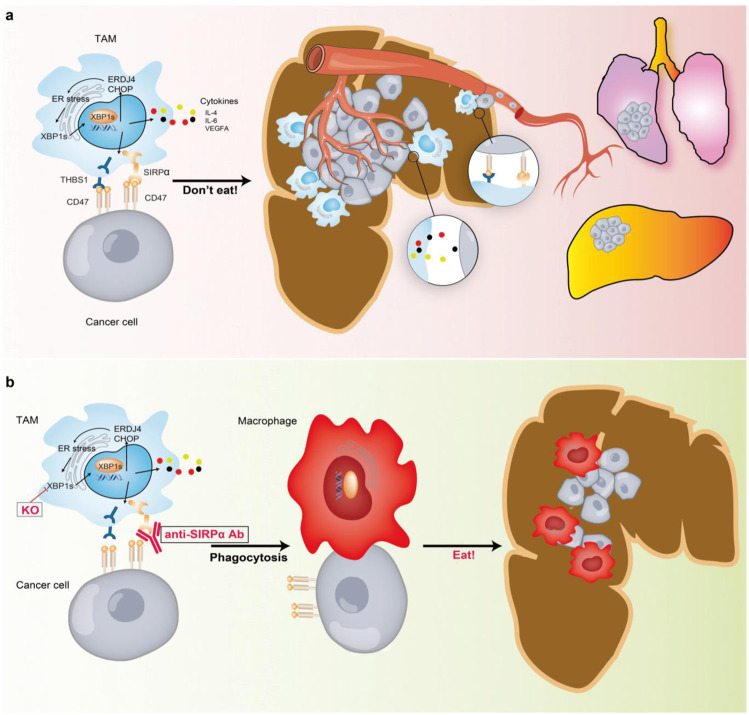
Scheme depicting the contribution of XBP1 in TAMs to colon cancer progression. **a** In the tumor microenvironment, activation of UPR/ER-XBP1 signaling in TAMs induced the production of cytokines, which inhibited macrophage phagocytosis of tumor cells via the disruption of self-recognition. Therefore, TAMs promote the metastasis of colorectal cancer. **b** Disabling UPR/ER-XBP1 signaling or treatment with anti-SIRPα antibodies may enhance anti-cancer capacity in a harsh tumor microenvironment

XBP1 activation is detected in TAMs of colon cancer patients and AOM-DSS mouse model in our study. XBP1 activation enhances the pro-tumor function of TAMs. Targeting XBP1 inhibits the ability of pro-tumor cytokines, such as IL-6 and VEGFA, as well as promotes tumor growth and metastasis. Concurrently, XBP1 inhibition downregulates the expression of membrane proteins, SIRPα and THBS1, blocking the “don’t eat me” recognition signal, enhancing the phagocytosis function (Fig. 7). Although more evidence of the therapeutic effect of XBP1 signaling in TAMs should be accumulated, our results suggest that XBP1 pathway in TAMs shows potential as a novel therapeutic target in CRC treatment.

## Materials and methods

### Tissues, mice, and cell lines

Human CRC specimen samples were procured from the Surgical Pathology Unit at the Chinese Academy of Medical Sciences Cancer Hospital (Beijing, China). Informed consent was obtained from all subjects, and this study was approved by the ethical committees of the Cancer Hospital, Chinese Academy of Medical Sciences. The clinical backgrounds for these patients are shown in Supplementary Tables S1–S3. Peripheral blood was collected from patients and centrifuged with Ficoll density gradient, following which the middle layer cells were gathered for fluorescence-activated cell sorting (FACS) analysis. Mice were housed at our Institutional Animal Care unit. All animal experiment protocols were approved by the ethical committee of the Chinese Academy of Medical Sciences, Cancer Hospital.

THP-1 (TIB-202, RRID:CVCL_0006), HCT116 (CCL-247, RRID:CVCL_0291) and DLD1 (CCL-221, RRID:CVCL_0248) cells were from ATCC. Ana-1 (EP-CL-0023, RRID:CVCL_0142) and CT26 cells (EP-CL-0071, RRID:CVCL_7254) were from Elabscience (Texas, USA). All the cells were maintained in RPMI 1640 (Bioroc™, China), supplemented with 10% heated-inactivated fetal bovine serum, 100 UI/mL penicillin, and 100 μg/mL streptomycin.

### AOM-DSS-induced CRC model

On day 1, C57BL/6J mice were injected i.p. with a single dose of AOM (Sigma-Aldrich Corp., St. Louis, MO, USA) at 2.5 mg/kg body weight as previously described.^51^ One week later, these mice were administered three cycles of 2.5% DSS for 5 days in sterile water, followed by one week in regular sterile water. Starting at week 8, the mice received different treatments (CEL clodronate liposome or anti-SIRPα). Tumor induced mice were randomized into respective treatment groups. CEL or PBS was administered twice a week (i.p., 12.5 μg). Anti-SIRPα (Bio X Cell, clone P84) or IgG were administered twice a week (i.p.). The mice were sacrificed 13 weeks later, colons removed and cut longitudinally. The number of tumors in colon of each mouse was counted. Portions of the distal colon were fixed with 4% paraformaldehyde overnight at 4 °C and embedded in paraffin.

### Tumor-associated macrophage induction and lentivirus

hTAMs (CD14^+^ CD11b^+^ CD206^+^) and PBMs (CD14^+^ CD11b^+^) in single-cell suspensions were sorted using FACSCalibur flow cytometry (Becton, Dickinson and Co., San Jose, CA, USA). Anti-human CD14 (clone 63D3, labeled with Percp-cy5.5), CD11b (clone ICRF44, labeled with APC) and CD206 (clone 15-2, labeled with PE) were purchased from BioLegend (San Diego, CA). mTAMs (CD14^+^ CD11b^+^ CD206^+^) from single-cell suspensions of AOM-DSS-induced colon tumors were sorted. APC-anti-mouse CD11b (clone M1/70) was from eBioscience (CA, USA). Percp-cy5.5-anti-mouse CD14 (clone Sa14-2) and FITC-anti-mouse CD206 (clone C068C2) were from BioLegend. Control spleen macrophages (sMs) from spleens of naive or CRC-bearing mice were FACS sorted by CD14 and CD11b. In all cases, BMDMs were generated from bone marrow in C57BL/6J mice via incubation in media supplemented with 20 ng/mL recombinant granulocyte macrophage colony-stimulating factor (M-CSF) (R&D, MN, USA). BMDMs were cultured in DMEM supplemented with 10% FBS at 37 °C in 5% CO2 and harvested on day 7 of expansion and used for subsequent CM induce.^52^ After 72 h, cells were detected for FACS to confirm CD206^+^ macrophages (miTAMs). PBMC-derived macrophages were generated from peripheral blood mononuclear cell and were prepared to incubate with conditioned medium for 72 h. CD206^+^ macrophages were considered to be hiTAMs.

THP-1 (M0) cells were generated from THP-1 via incubation with LPS (10 ng/mL) for 96 h. THP-1 (M2) cells were generated from THP-1 (M0) incubated with IL-4 (20 ng/mL) for 48 h. TAMs (THP-1 CM) were generated from THP-1 (M2) cells induced via exposure to condition medium from HCT116 cells for 72 h. SgXBP1 hTAMs were inoculated with lentivirus LentiCRISPR v2 sgRNA targeting *XBP1* (5’-GGGCATTTGAAGAACATGAC-3’), and sgCon hTAMs were inoculated with a nontargeting sgRNA (5’-ACGGAGGCTAAGCGTCGCAA-3’).

TAMs (Ana-1 CM) were generated from Ana-1 cell induced by exposure to condition medium from CT26 cells for 72 h. SgXbp1 mTAMs were inoculated with lentivirus LentiCRISPR v2 sgRNA targeting *Xbp1* (5’-ACTTGTCCAGAATGCCCAAA-3’), and sgCon mTAMs were inoculated with a nontargeting sgRNA (5’-GCGAGGTATTCGGCTCCGCG-3’).

### RNA-seq and macrophage transcriptional profile

TAMs were sorted from human CRC tumor and AOM-DSS-induced CRC mice single-cell suspensions. Total RNA was isolated using RNeasy Mini Kit (Qiagen, Germany) and RNA quality and integrity confirmed via an Agilent Bioanalyzer 2100. Next, mRNA libraries were generated and sequenced at the WuXiNextCODE Genomics (Shanghai) Co., Ltd.

### Immunofluorescence staining

Tumor tissues were fixed in 4% paraformaldehyde and immunofluorescence was performed on 5-μm-thick paraffin sections after heat-induced antigen retrieval. The following primary antibodies were used: CD68 (1:200, Abcam, ab53444); CD206 (1:200, Proteintech, 60143-1-Ig), XBP1 (1:100, Abcam, ab37152), F4/80 (1:200, Abcam, ab6640) and EPCAM (1:800, CST, #2929). Subsequently, we incubated the slides with fluorescent secondary antibodies (Fluorescein (FITC)-conjugated Goat Anti-Rabbit IgG(H + L), 1:1000, Proteintech, SA00003-2; Rhodamine (TRITC)-conjugated Goat Anti-Rat IgG (H + L), 1:1000, Proteintech, SA00007-7; Alexa Fluor® 647 Conjugate Goat Anti-mouse IgG (H + L), 1:1000, Cell Signaling Technology, #4410), DAPI (Thermo Scientific, #62247) and Opal 7-Color Manual IHC Kit (Akoya Biosciences, NEL811001KT) for multilabel immunofluorescence analysis.

### Phagocytosis assay

sgCon or sgXBP1 TAMs (5 × 10^4^) were co-cultured with RFP-labeled DLD1 or CT26 cancer cells (2.5 × 10^5^) in 24-well tissue-culture plates. Living cells were assessed by Ultra*VIEW* VoX (PerkinElmer) for 8 h at 37 °C and wells were washed thoroughly with DMDM three times and analyzed using Volocity software (PerkinElmer). Phagocytic index was calculated using as described, phagocytic index = number of ingested cells/(number of macrophages/100).^53^

### Orthotopic xenograft CRC mouse model, liver metastasis, and lung metastasis model

For the orthotopic xenograft CRC mouse model, CT26 (2 × 10^5^ cells) were mixed with the indicated miTAMs (2 × 10^5^ cells) and were co-injected into the wall of the cecum in NOD/SCID mice (*n* = 5 per group). After 4 weeks, all the mice were sacrificed. Colons and livers, were harvested to assess the tumor burden. HE staining demonstrating the histology of tumors formed in the livers was observed using the ImageScope software as described.^54^

NOD/SCID mice were injected intrasplenically with CT26-luciferase cells (1 × 10^5^ cells, stably expressed firefly luciferase) and miTAMs (1 × 10^5^ cells) by splenectomy 3 min after injections, for the liver metastasis model.^55^ On day 15 following model establishment, 200 µL of 15 mg/mL luciferin (PerkinElmer) was intraperitoneally injected into anesthetized mice, and bioluminescence was examined 10 min after injection using an IVIS Lumina system (PerkinElmer). Bioluminescence was quantified based on the photon flux ratio. HE staining demonstrating the histology of tumors formed in the livers with the ImageScope software.

For the induction of lung metastasis, NOD/SCID mice were injected intravenously (i.v.) with CT26 cells (1 × 10^5^ cells) and miTAMs (1 × 10^5^ cells) in 100 μL PBS, and were sacrificed 20 days later. To quantify the metastatic burden, pulmonary metastatic nodule numbers were calculated. HE staining demonstrating the histology of tumors formed in the lungs was observed using the ImageScope software.

### Cytokine antibody array

CM from sgCon TAMs and paired sgXBP1 TAMs were tested for cytokine secretion using selected cytokine antibody arrays, following the manufacturer’s instructions (Ray Biotech, Inc, Norcross, GA, USA).

### ChIP-qPCR

BMDM cells were separated from bone marrow of C57BL/6J mice and trained to produce TAMs using condition medium from CT26 cells. Cells were washed twice in cold PBS supplemented with protease inhibitors (Roche), fixed for 15 min in 1% formaldehyde (Sigma-Aldrich) at room temperature and quenched by adding 125 mmol/L glycine for 5 min. ChIP was performed with XBP1 antibodies (sc-7160; Santa Cruz Biotechnology, CA). All primers used are described in Supplementary Table S3.

### Generation of macrophages-specific Cre-dependent Cas9 mice

Conditional Cas9 mice Lox-stop-Lox-Cas9 (C57BL/6-Gt(ROSA)26Sor^*tm1(CAG-LNL-Cas9)Smoc*^) were obtained from the Shanghai Model Organisms Center, Inc. and crossed with bone marrow cells-specific Lyz2-Cre mice (C57BL/6*-Lyz2*^*em1(2A-CreERT2-WPRE-pA)Smoc*^) in order to generate experimental Lyz2-Cre-LSL-Cas9 mice. The following primers were used to genotype Cas9: Cas9_Common-S 5′-TCCCGACAAAACCGAAAATCTGTGG-3′; Cas9_Wild-AS 5′-GGGGCGTGCTGAGCCAGACCTCCAT-3′; Cas9_Mut-AS 5′- TGCATCGCATTGTCTGAGTAGG-3′) and Lyz2-Cre (5′-CTTGGGCTGCCAGAATTTCTC-3′ for common-S; 5′-CCCAGAAATGCCAGATTACG-3′ for Wild-type-AS; and 5′-TTACAGTCGGCCAGGCTGAC-3′ for Cre-AS in Lyz2-Cre mice as previously described.^56,57^

### SgRNA-Xbp1 Adeno-associated virus (AAV2) production

Effective sgRNAs targeting mouse XBP1 were first validated using lentiviral vectors, and adenovirus pX601-AAV-CMV::NLS-SaCas9-NLS-3xHA-bGHpA;U6::BsaI-sgRNA (Addgene) targeting (AAV2-sgXbp1) or nontargeting XBP1 (AAV2-sgControl) were generated. Then, 293FT cells were transfected using Lipofectamine 2000 (Invitrogen, Carlsbad, CA, USA) with the AAV vector along with helper packaging vectors. Two days later, the cells and media were collected and subjected to four freeze-thaw cycles by alternating between an ethanol dry ice bath and 37 °C. Cell debris was removed by centrifugation and the supernatant was collected, passed through a 0.45 µm filter, aliquoted, and frozen at −80 °C until further use.

### Quantification and statistical analysis

All experiments were repeated at least twice, where the results of repeats were similar. Animal experiments used between three and six mice per group. Statistical significance was set at *P* < 0.05. All statistical analyses were conducted using Graph Pad Prism 7.0. Differences between the means of experimental groups were analyzed using unpaired Student’s *t*-test or ANOVA analysis. Error bars represent SEM of independent samples assayed within representative experiments. Survival rates were compared using the log-rank test. All survival experiments used at least six mice per group. This number provides a 5% significance level and 95% power to detect differences in the survival of 20% or greater.

## Supplementary information

supplementary- clean

## Data Availability

All the datasets used and/or analyzed during this study are available from the corresponding author on reasonable request.
