# Supplementary material for: XBP1 regulates the protumoral function of tumor-associated macrophages in human colorectal cancer
Source: Signal Transduct Target Ther. 2021 Oct 20;6:357. doi: 10.1038/s41392-021-00761-7 (PMC8526672; doi:10.1038/s41392-021-00761-7)
Supplement: Supplementary file 1 — supplementary- clean [file 41392_2021_761_MOESM1_ESM.docx]

Supplementary Materials for

XBP1 regulates the protumoral function of tumor-associated macrophages in human colorectal cancer

Yahui Zhao^1^, Weina Zhang^1^, Miaomiao Huo^1^, Peng Wang^2^, Xianghe Liu^1^, Yu Wang^1^, Yinuo Li^1^, Zhixiang Zhou^2^, Ningzhi Xu^1^*, Hongxia Zhu^1^*

^1^ State Key Laboratory of Molecular Oncology, National Cancer Center/National Clinical Research Center for Cancer/Cancer Hospital, Chinese Academy of Medical Sciences and Peking Union Medical College, Beijing 100021, China

^2^ Department of Colorectal Surgery, National Cancer Center/National Clinical Research Center for Cancer/Cancer Hospital, Chinese Academy of Medical Sciences and Peking Union Medical College, Beijing, 100021, China

Correspondence to: Hongxia Zhu, Ph.D., State Key Laboratory of Molecular Oncology, National Cancer Center/National Clinical Research Center for Cancer/Cancer Hospital, Chinese Academy of Medical Sciences and Peking Union Medical College, Beijing 100021, China. Tel.: +861087787340. E-mail: drhxzhu@cicams.ac.cn. Ningzhi Xu, M.D., State Key Laboratory of Molecular Oncology, National Cancer Center/National Clinical Research Center for Cancer/Cancer Hospital, Chinese Academy of Medical Sciences and Peking Union Medical College, Beijing 100021, China. Tel.: +861087788487. E-mail: xuningzhi@cicams.ac.cn

**This file includes:**

Materials and Methods

Supplementary Figures S1 to S7

Supplementary Tables S1 to S3

**Supplementary Materials and Methods**

**Isolation of human and mouse macrophages**

Human patient CRC-associated macrophages (hTAMs) (CD14^+^CD11b^+^CD206^+^) and PBMs (CD14^+^CD11b^+^) in single-cell suspensions were sorted using FACSCalibur flow cytometry (Becton, Dickinson and Co., San Jose, CA, USA). Anti-human CD14 (clone 63D3, labeled with Percp-cy5.5), CD11b (clone ICRF44, labeled with APC) and CD206 (clone 15-2, labeled with PE) were purchased from BioLegend (San Diego, CA). Mouse tumor-associated macrophages (mTAMs) (CD14+CD11b+CD206+) from single-cell suspensions of AOM-DSS-induced colon tumors were sorted. APC-anti-mouse CD11b (clone M1/70) was from eBioscience (CA, USA). Percp-cy5.5-anti-mouse CD14 (clone Sa14-2) and FITC-anti-mouse CD206 (clone C068C2) were from Biolegend. Control spleen macrophages from spleens of naive or CRC-bearing mice were FACS sorted. In all cases, BMDMs were generated from bone marrow in C57BL/6J mice via incubation in media supplemented with 20 ng/mL recombinant granulocyte macrophage colony-stimulating factor (M-CSF) (R&D, MN, USA). Cells were harvested on day 15 of expansion and used directly for subsequent *in vitro* or *in vivo* functional assays.

**Boyden chamber**

For the transwell assays, inserts were coated with Matrigel for the invasion assay, respectively. Next, 5 × 10^4^ CT26 cells were seeded into the upper chamber and starved for 12 h prior to experiments. TAMs were seeded into the lower chamber 12 h prior to experiments.

**ELISA**

Conditioned medium from sgCon TAMs and paired sgXBP1 TAMs were collected 96 h later and centrifuged to remove dead cells. The supernatants were then analyzed using IL-6 and VEGFA-specific enzyme-linked immunosorbent assay (ELISA) kit (eBioscience). Standards were assayed in duplicate and the values were normalized to the total protein content.

**Supplementary Figures**

**
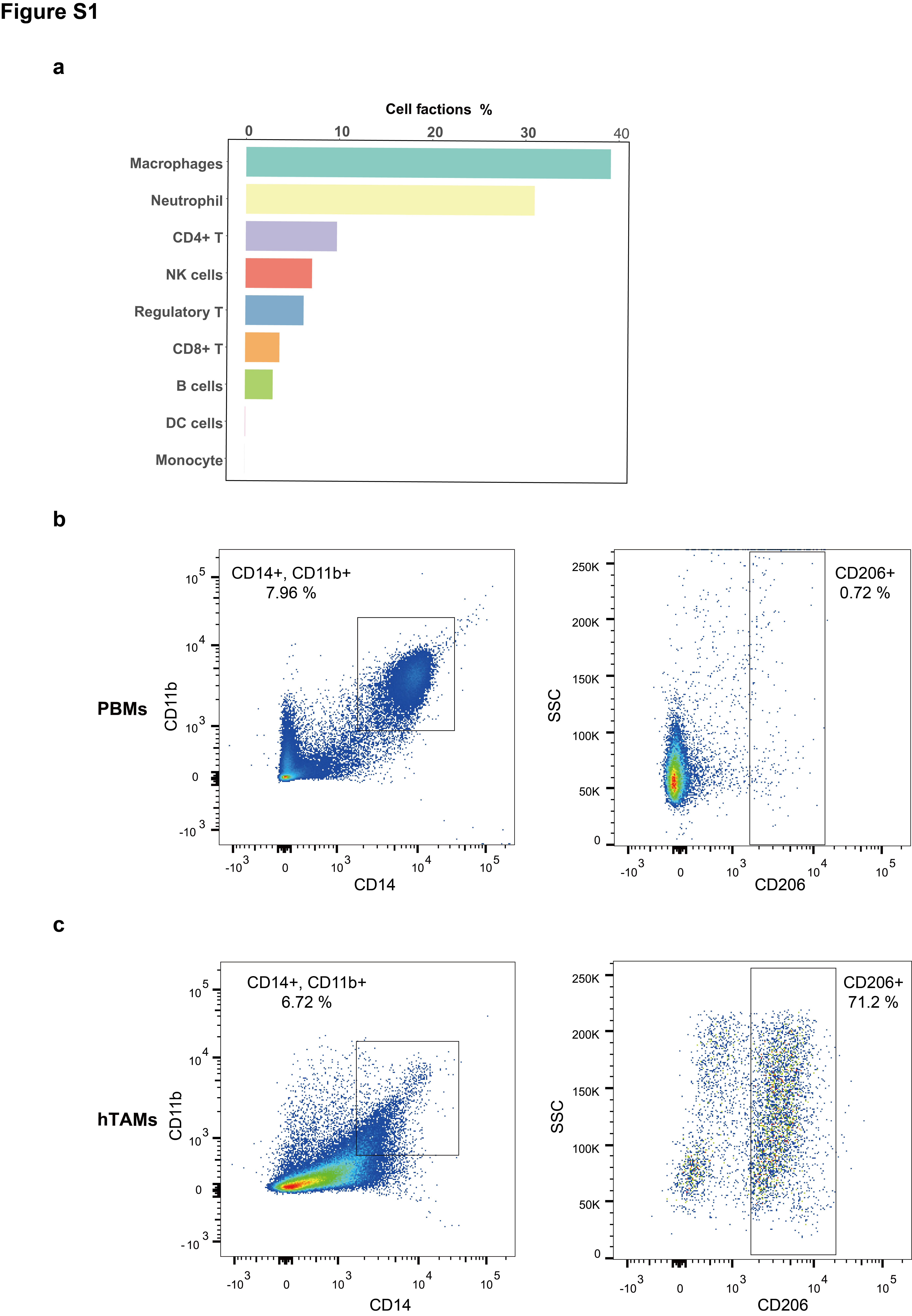
**

**Fig. S1 Isolation and analysis of TAM populations of human origin.**

(**a**) Infiltration of different immune cells in CRC according to the Cancer Genome Atlas (TCGA) RNA-seq. (**b**) Gating strategy to isolate control PBMs (CD14^+^, CD11b^+^) from blood. (**c**) Gating strategy used to isolate human CRC hTAMs (CD14^+^, CD11b^+^ and CD206^+^) in solid tumors.

**
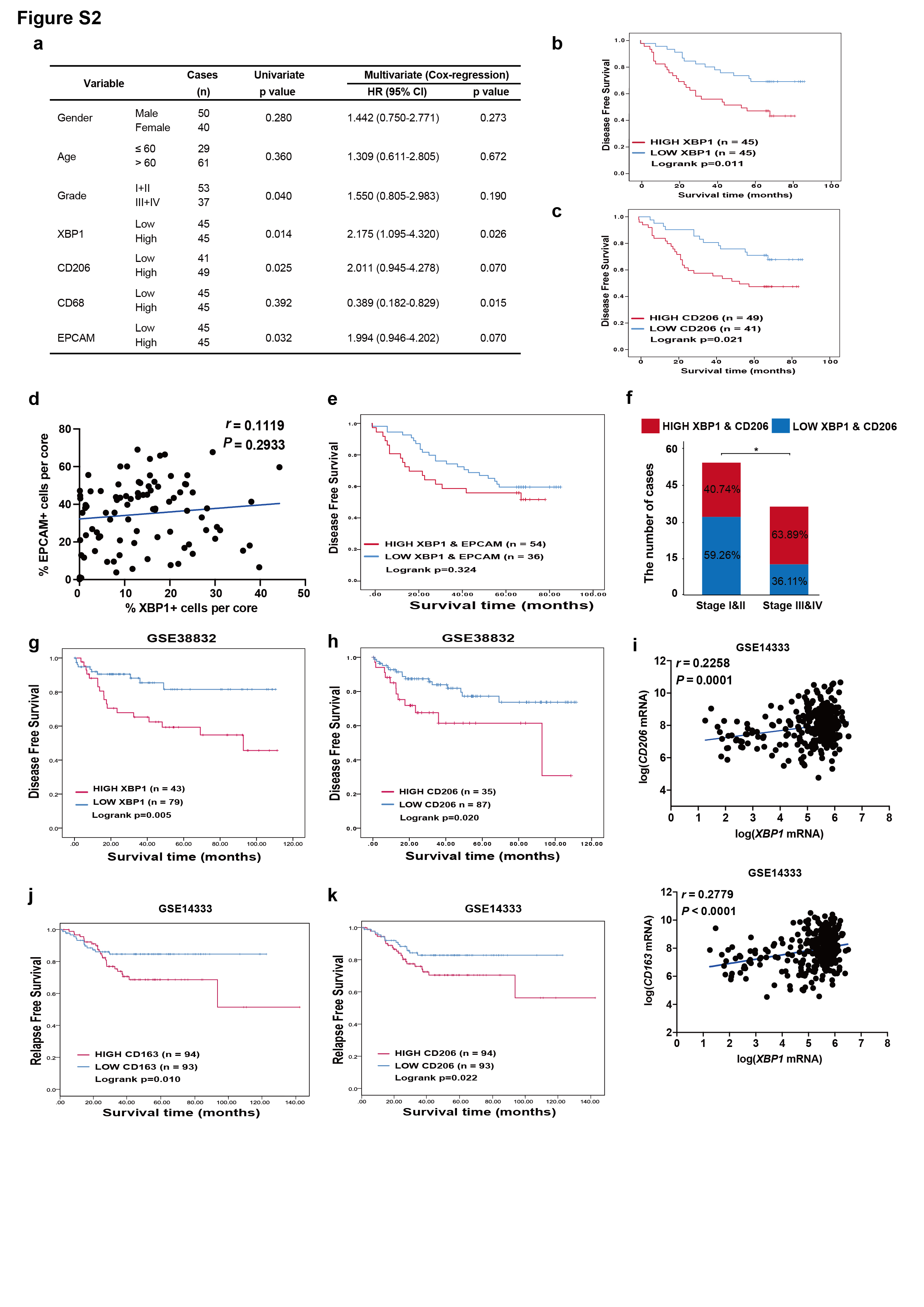
**

**Fig. S2 Correlation between XBP1 and prognosis of CRC patients.**

(**a**) Univariate and multivariate analysis predicts risk factors of poor survival for CRC patients. **(b, c)** Kaplan-Meier survival curves for 90 CRC patients with or without high XBP1^+^ (**b**) or CD206^+^ (**c**) cells. **(d)** Quantification of the percentage of XBP1^+^ or EPCAM^+^ cells among the total number of cells in each individual core in the TMA, as determined by PerkingElmer inForm^TM^ system. Correlation of percentage of XBP1^+^ cells with EPCAM^+^ cells in CRC patients. r, Spearman’s rank correlation test. **(e)** Kaplan-Meier survival curves for 90 CRC patients with or without high XBP1^+^EPCAM^+^ cells. (**f**) Representative the statistical analysis of TMA-based multilabel immunofluorescence analysis, showing the higher expression of XBP1^+^ CD206^+^ TAMs in tumor tissue is correlated to TNM stage. * =  *P*<0.05; Fisher Exact Test. **(g, h)** Kaplan-Meier survival curves for CRC metastasis patients with or without high expression of XBP1 (**g**) and CD206 (**h**) in GEO online database (GSE38832). The optimal survival cut point was determined by X-Tile statistical software. **(i)**The correlation of *XBP1* and *CD163* in tumor tissues (*CD206*, upper panel and *CD1636*, lower panel). The association was analyzed using coefficient measures of linear relationships in the public GEO database (GSE14333). (**j, k**) Kaplan-Meier survival curves for CRC patients with expression of *CD163* or *CD206* in GEO online database (GSE14333). The optimal survival cut point was determined by X-Tile statistical software.

**
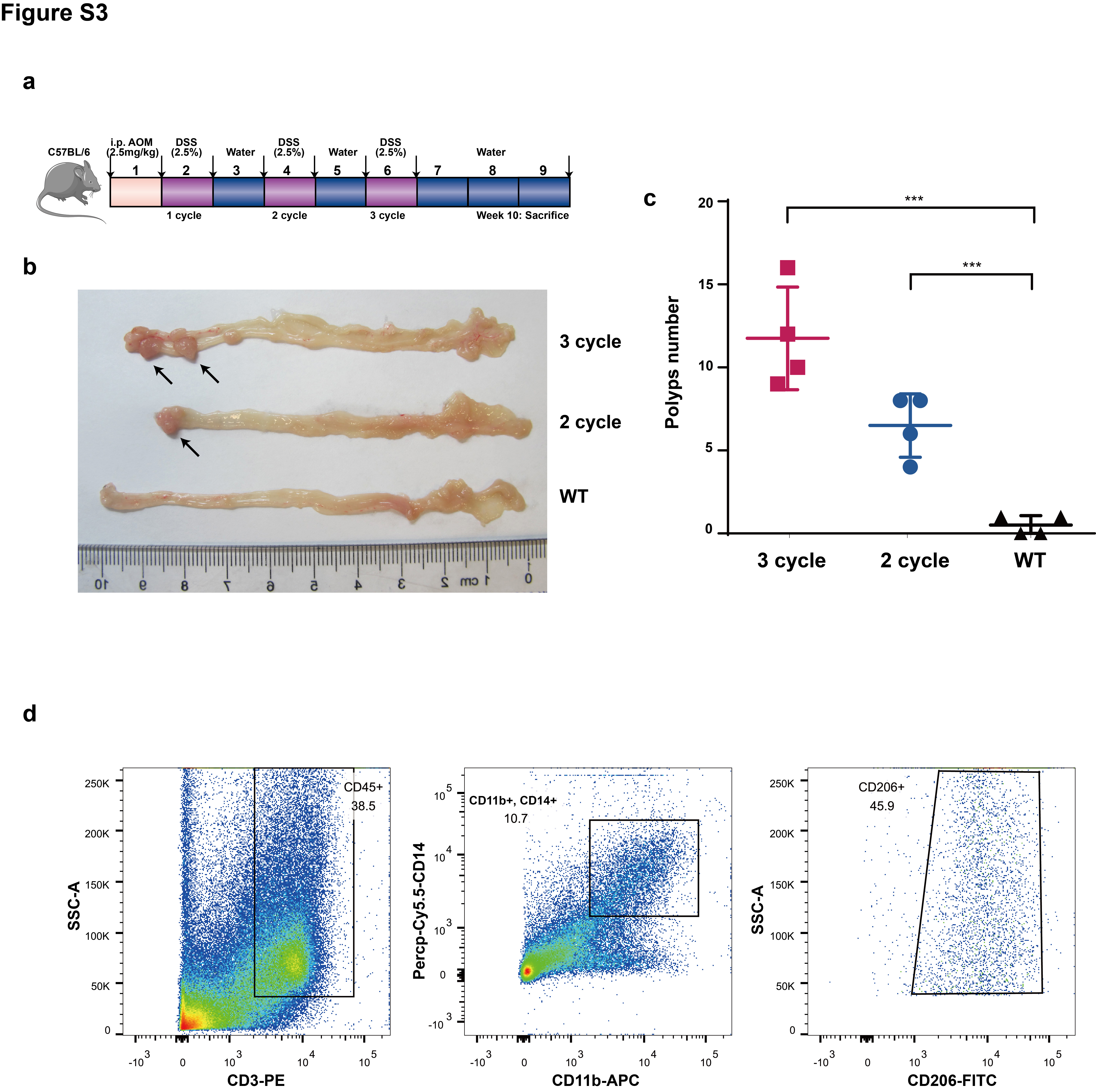
**

**Fig. S3 TAMs infiltrated in AOM-DSS-induced colon cancer.**

(**a**) Schematic overview of the experimental design. Colitis-associated colon cancer was induced in C57BL/6 mice by AOM/DSS treatment (2 cycle n= 4, 3 cycle n = 4). The untreated control mice (n = 4) were littermates of similar age. *** = *P*<0.001; ANOVA analysis. (**b**) Representative pictures of the whole colons. The arrowhead indicated macroscopic polyps. (**c**) Mean macroscopic polyp number in the whole colon. (**d**) Gating strategy used to isolate AOM-DSS-induced colon cancer TAMs present in solid tumors.

**
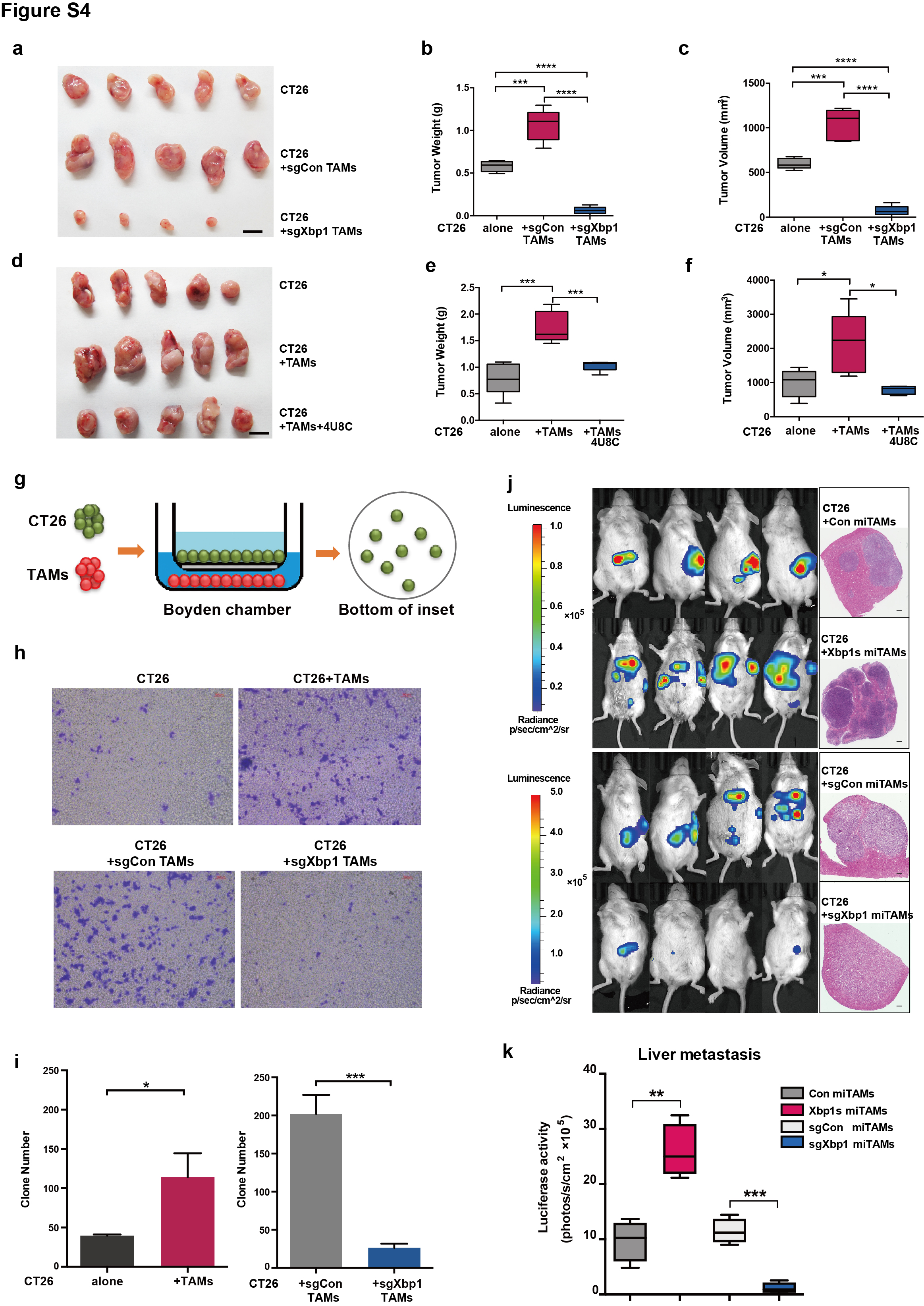
**

**Fig. S4 The function of XBP1 activation in TAMs.**

(**a-c**) Effect of Xbp1 knockout in TAMs. Colorectal cancer cells (CT26) were implanted in the flank, either alone or in combination with sgCon or XBP1-deficient (sgXbp1) TAMs (Ana-1 CM). Tumors were resected and measured 14 d later. (**a**) Micrographs of tumors, (**b**) tumor weights, and (**c**) tumor volumes. *** = *P*<0.001, **** = *P*<0.0001; Error bars represent SEM; ANOVA analysis. (**d-f**) Effect of targeted inhibition of macrophage XBP1 activation on tumor proliferation. CT26 were implanted in the flank, either alone or in combination with TAMs, treated with or without 4U8C. Tumors were resected and measured 14 d later. (**d**) Pictures of tumors, (**e**) Tumor weights and (**f**) Tumor volumes; * = *P*<0.05, *** = *P*<0.001. Error bars represent SEM; ANOVA analysis. (**g**) Boyden chamber assay. CRC tumor cells (CT26) and TAMs (Ana-1 CM) were co-cultured following the diagram as indicated. (**h**) The migration capacity of CT26. The migration capacity of CT26, either alone or co-cultured with TAMs (Ana-1 CM). Mean cell numbers migrating to the lower side of the membrane (upper panel) **.** The migration capacity of CT26 co-cultured with sgCon TAMs (Ana-1 CM) or sgXbp1 TAMs (Ana-1 CM) were visualized using the Boyden chamber assay (lower panel). (**i**) Statistical analysis of the cell numbers that migrated to the lower side of the membrane in (**h**)**.** * = *P*<0.05, *** = *P*<0.001; t-test. (**j**) Representative images of liver metastases induced by splenic injection in NOD/SCID mice with CT26-Luciferase. CT26-luciferase cells were mixed with Con miTAMs; Xbp1s miTAMs; sgCon miTAMs; and sgXbp1 miTAMs. HE staining demonstrating the histology of tumors formed in the liver; scale bar, 500 μm. (**k**) Quantification of the tumor luminescence signal; ** = *P*<0.01, *** = *P*<0.001; t-test.

**
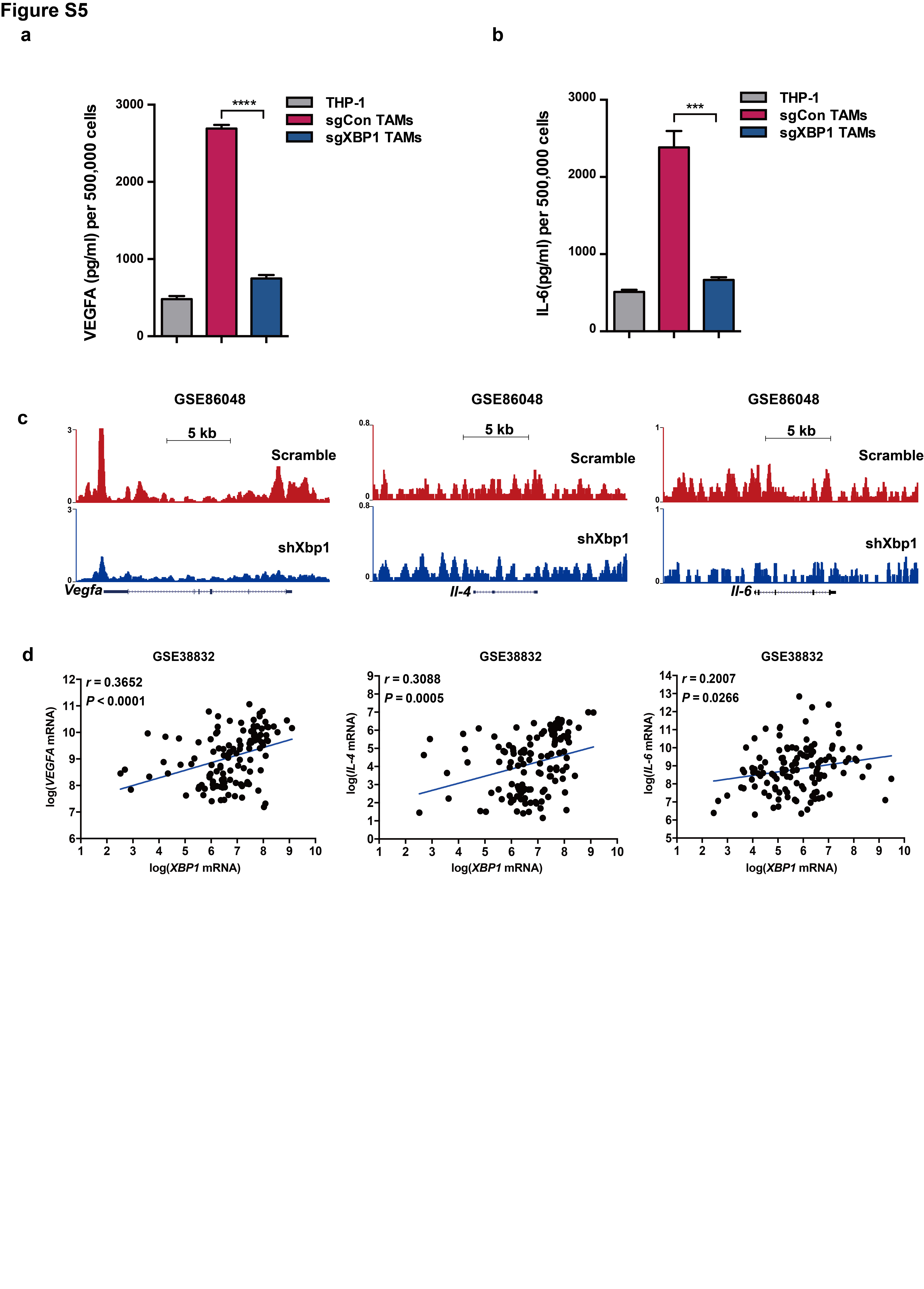
**

**Fig. S5 The effect of XBP1 on cytokine production.**

(**a, b**) Concentration of VEGFA (**a**) and IL-6 (**b**) in THP-1, sgCon, or sgXBP1 TAMs (THP-1 CM) supernatants were measured via ELISA assay. *** = *P*<0.001, **** = *P*<0.0001; t-test. (**c**) Track view of *Vegfa*, *Il-4*, and *Il-6* ChIP-seq density upon silencing of Xbp1 in the ChIP-seq online database (GSE49955). (**d**) Correlation of *XBP1* with *VEGFA*, *IL-4* and *IL-6* in CRC patients. The association was analyzed using coefficient measured linear relationships in the public GEO database (GSE38832).


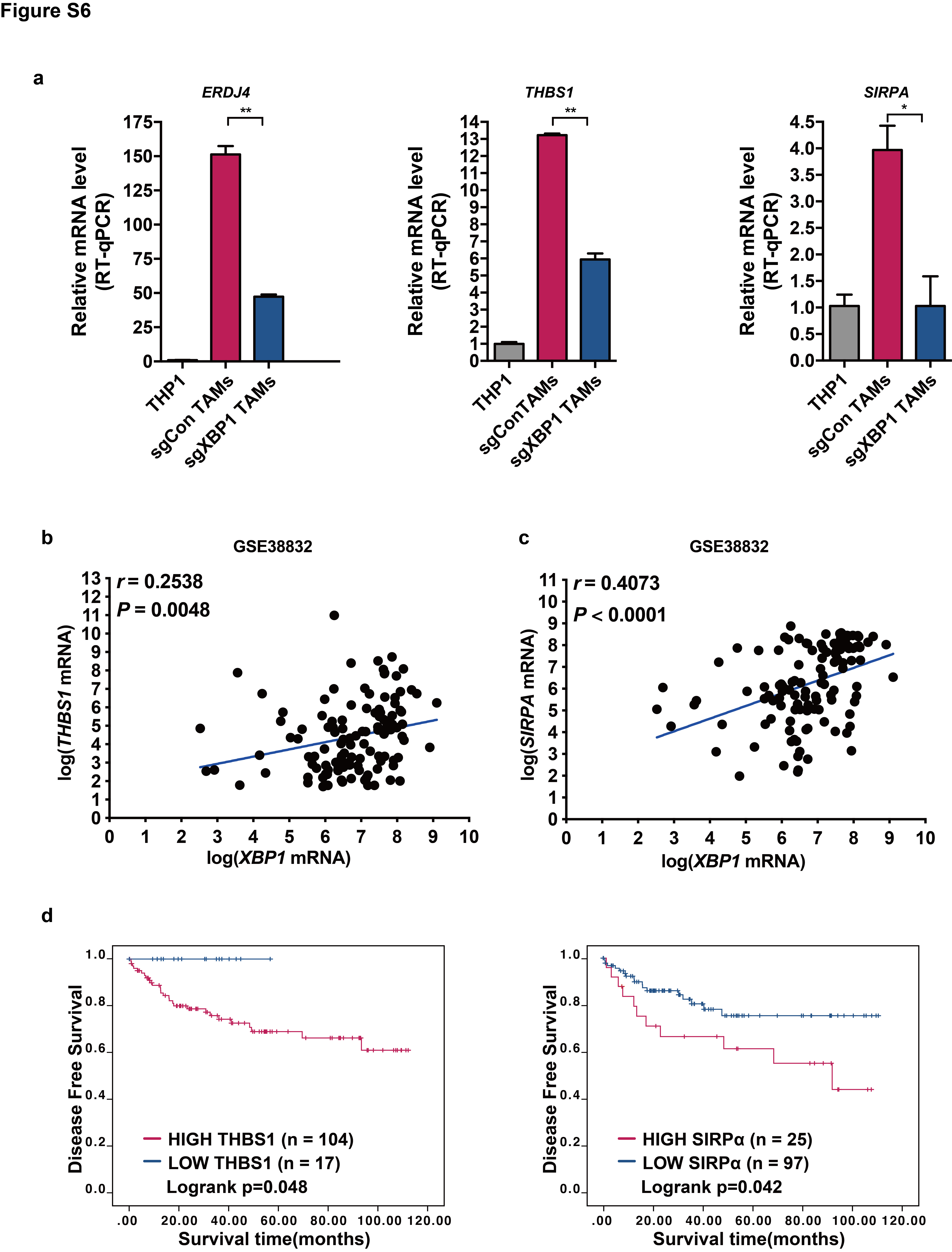


**Fig. S6 The correlation between *XBP1* and *THBS1* and *SIPR*α.**

(**a**) Relative mRNA levels of *XBP1* and phagocytosis-associated genes in THP-1 (grey), scramble TAMs (THP-1 CM, magenta) and sgXBP1 TAMs (THP-1 CM, blue) validated by RT-qPCR. Bars represent mean ± SD of three experimental replicates. * = *P*<0.05, ** = *P*<0.01. *P* values were determined using t-test. (**b, c**) The correlation between *XBP1* and *THBS1* (**b**) and *XBP1* and *SIPRα* (**c**) in CRC patients. The association was analyzed using coefficient measures of linear relationships in the public GEO database (GSE38832). (**d**) Kaplan-Meier survival curves for CRC patients expressing THBS1 or SIPRα in GEO online database (GSE14333). The optimal survival cut point was determined via X-Tile statistical software.


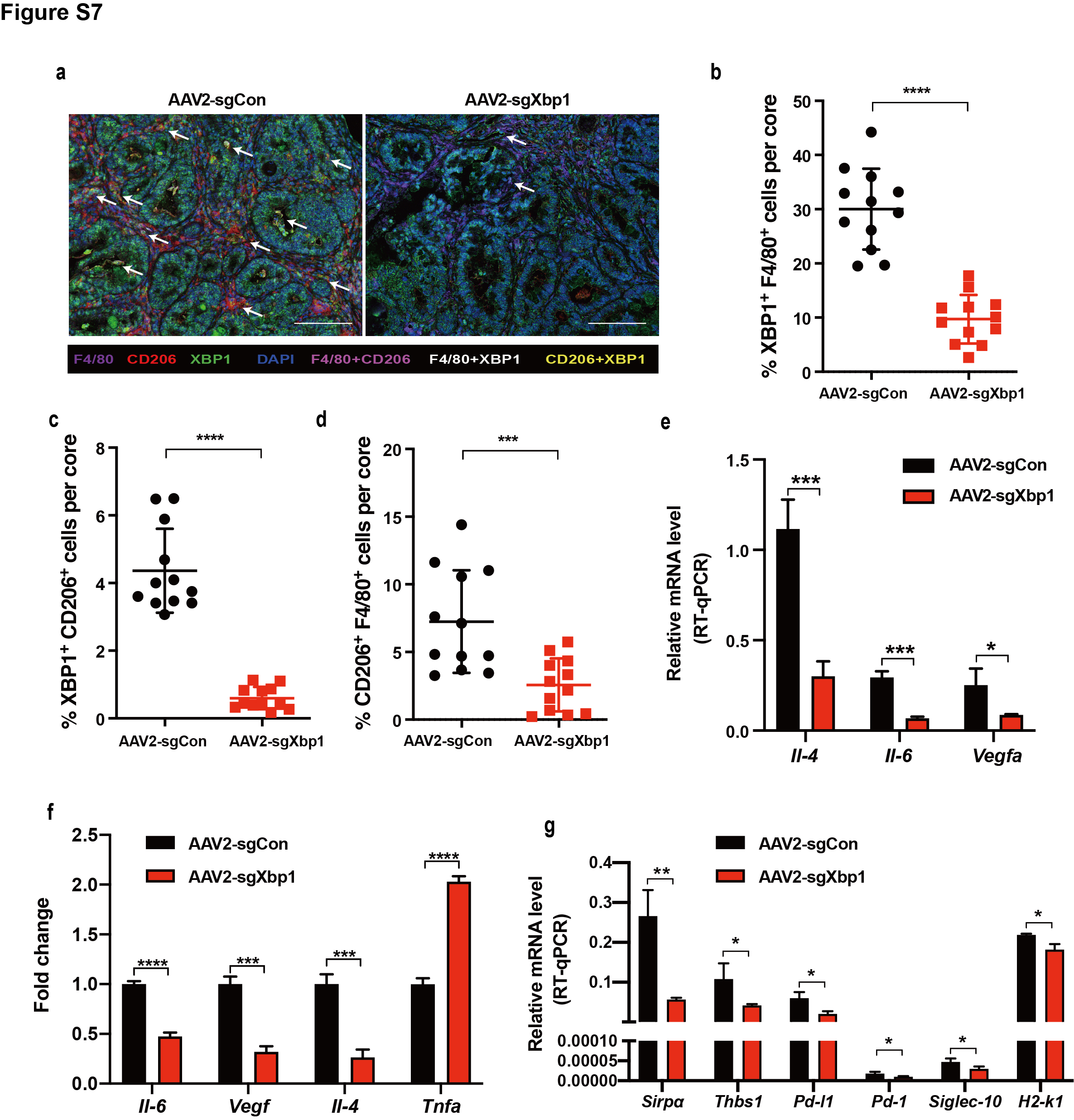


**Fig. S7 Conditional deletion of XBP1 in TAMs inhibited “M2 like” TAMs markers, cytokines or recognition signals in vivo.**

(**a**) F4/80, CD206, and XBP1 immunofluorescence in colon sections from AAV2-sgCon and AAV2-sgXbp1 AOM-DSS mice. White arrows denote phagocytic events. Scale bar = 40 μm. **(b-d)** Quantification of the percentage of XBP1^+^ F4/80^+^ cells (**b**), XBP1^+^ CD206^+^ cells (**c**), and CD206^+^ F4/80^+^ cells (**d**) among the total number of cells in each individual core from AAV2-sgCon and AAV2-sgXbp1 AOM-DSS mice, as determined by PerkingElmer inForm^TM^ system. *** = *P*<0.001, **** = *P*<0.0001; t-test. (**e**) Relative mRNA levels of tumor-promoting cytokines (*Il-4*, *Il-6* and *Vegfa*) in AAV-sgCon TAMs (black) and AAV-sgXbp1 TAMs (red) validated by RT-qPCR. **(f)** Concentration of IL-6, VEGFA, IL-4 and TNFα for the TIF samples of AAV2-sgCon mTAMs (black) and AAV2-sgXbp1 mTAMs (red) validated via ELISA assay. *** = *P*<0.001, **** = *P*<0.0001; t-test. **(g)** Relative mRNA levels of recognition signals (*Sirpa*, *Thbs1*, *Pd-l1*, *Pd-1*, *Siglec-10* and *H2-k1*) in AAV2-sgCon mTAMs (black) and AAV2-sgXbp1 mTAMs (red) validated by RT-qPCR. * = *P*<0.05, *** = *P*<0.001; t-test.

| **Sample ID** | **Gender** | **Age at diagnosis** | **Stage (TNM)** | **LNM** | **Site** | **Differentiation** |
| --- | --- | --- | --- | --- | --- | --- |
| CRC-01 | Female | 61 | I | Non-metastasis | Colon | Moderately |
| CRC-02 | Female | 54 | II | Non-metastasis | Rectum | Moderately |
| CRC-03 | Female | 62 | II | Non-metastasis | Colon | Moderately |
| CRC-04 | Male | 60 | II | Non-metastasis | Colon | Moderately |
| CRC-05 | Male | 51 | II | Non-metastasis | Colon | Moderately |
| CRC-06 | Female | 65 | II | Non-metastasis | Rectum | Moderately |
| CRC-07 | Male | 60 | II | Non-metastasis | Rectum | Moderately |
| CRC-08 | Female | 47 | II | Non-metastasis | Rectum | Moderately |
| CRC-09 | Male | 85 | I | Non-metastasis | Colon | Moderately |
| CRC-10 | Male | 71 | III | Non-metastasis | Colon | Moderately |
| CRC-11 | Male | 67 | II | Non-metastasis | Rectum | Poorly |
| CRC-12 | Female | 49 | II | Non-metastasis | Colon | Poorly |
| CRC-13 | Female | 73 | II | Non-metastasis | Rectum | Moderately |
| CRC-14 | Female | 63 | I | Non-metastasis | Rectum | Moderately |
| CRC-15 | Male | 74 | III | Metastasis | Colon | Poorly |
| CRC-16 | Male | 75 | IV | Metastasis | Rectum | Moderately |
| CRC-17 | Female | 51 | III | Metastasis | Rectum | Moderately |
| CRC-18 | Female | 65 | III | Metastasis | Colon | Moderately |
| CRC-19 | Male | 45 | IV | Metastasis | Colon | Moderately |
| CRC-20 | Female | 70 | III | Metastasis | Colon | Moderately |
| CRC-21 | Male | 44 | III | Metastasis | Colon | Moderately |
| CRC-22 | Male | 63 | III | Metastasis | Rectum | Moderately |
| CRC-23 | Male | 55 | III | Metastasis | Colon | Poorly |
| CRC-24 | Female | 59 | III | Metastasis | Colon | Moderately |
| CRC-25 | Male | 54 | III | Metastasis | Rectum | Poorly |
| CRC-26 | Female | 67 | III | Metastasis | Rectum | Poorly |
| CRC-27 | Male | 54 | III | Metastasis | Colon | Poorly |

**Supplementary Tables**

**Table S1. Clinical features of the CRC patients.**

**Table S2. Summary of colorectal cancer patients demographic and clinical characteristics (N = 27).**

| **Factor** | **Variables** | **Proportion** |
| --- | --- | --- |
| **Age** | ≥ 60 | 16 (59.3%) |
|  | < 60 | 11 (40.7%) |
| **Gender** | Male | 14 (51.9%) |
|  | Female | 13 (48.1%) |
| **Stage** | I-II | 12 (44.4%) |
|  | III-IV | 15 (55.6%) |
| **LNM** | Non-metastasis | 14 (51.9%) |
|  | Metastasis | 13 (48.1%) |
| **Site** | Colon | 15 (55.6%) |
|  | Rectum | 12 (44.4%) |
| **Differentiation** | Moderately | 20 (74.1%) |
|  | Poorly | 7 (25.9%) |

**Table S3. Clinical features of the TMA multilabel immunofluorescence (MIF) in the 90 pairs of human CRC.**

| **Sample ID** | **Gender** | **Age at diagnosis** | **Stage (TNM)** | **LNM** | **DFS**  **(Event=1)** | **DFS. time**  **(Month)** |
| --- | --- | --- | --- | --- | --- | --- |
| ΤΜΑ-A01 | male | 40 | III | Metastasis | 1 | 7 |
| ΤΜΑ-A03 | Female | 59 | II | Non-metastasis | 0 | 86 |
| ΤΜΑ-A05 | male | 71 | II | Non-metastasis | 0 | 86 |
| ΤΜΑ-A07 | male | 82 | III | Metastasis | 1 | 26 |
| ΤΜΑ-A09 | male | 59 | III | Metastasis | 1 | 14 |
| ΤΜΑ-A11 | male | 62 | II | Non-metastasis | 0 | 85 |
| ΤΜΑ-A13 | male | 61 | III | Metastasis | 1 | 22 |
| ΤΜΑ-A15 | male | 75 | III | Metastasis | 0 | 84 |
| ΤΜΑ-A17 | Female | 41 | II | Non-metastasis | 0 | 84 |
| ΤΜΑ-B01 | male | 77 | III | Metastasis | 0 | 84 |
| ΤΜΑ-B03 | Female | 82 | II | Non-metastasis | 0 | 83 |
| ΤΜΑ-B05 | Female | 80 | III | Metastasis | 0 | 81 |
| ΤΜΑ-B07 | male | 78 | II | Non-metastasis | 1 | 42 |
| ΤΜΑ-B09 | Female | 66 | II | Non-metastasis | 0 | 81 |
| ΤΜΑ-B11 | Female | 76 | III | Metastasis | 1 | 44 |
| ΤΜΑ-B13 | male | 79 | III | Non-metastasis | 1 | 0 |
| ΤΜΑ-B15 | Female | 59 | IV | Metastasis | 1 | 34 |
| ΤΜΑ-B17 | male | 52 | III | Metastasis | 1 | 49 |
| ΤΜΑ-C01 | Female | 64 | III | Metastasis | 0 | 76 |
| ΤΜΑ-C03 | Female | 43 | I | Non-metastasis | 0 | 72 |
| ΤΜΑ-C05 | Female | 78 | II | Non-metastasis | 0 | 72 |
| ΤΜΑ-C07 | male | 75 | III | Metastasis | 1 | 7 |
| ΤΜΑ-C09 | Female | 63 | II | Non-metastasis | 0 | 70 |
| ΤΜΑ-C11 | male | 54 | III | Metastasis | 0 | 70 |
| ΤΜΑ-C13 | Female | 61 | II | Non-metastasis | 0 | 70 |
| ΤΜΑ-C15 | male | 57 | II | Non-metastasis | 1 | 23 |
| ΤΜΑ-C17 | male | 64 | II | Non-metastasis | 0 | 70 |
| ΤΜΑ-D01 | male | 84 | II | Non-metastasis | 0 | 70 |
| ΤΜΑ-D03 | male | 49 | III | Metastasis | 0 | 70 |
| ΤΜΑ-D05 | male | 45 | II | Non-metastasis | 0 | 70 |
| ΤΜΑ-D07 | male | 57 | II | Non-metastasis | 0 | 70 |
| ΤΜΑ-D09 | male | 77 | III | Metastasis | 0 | 70 |
| ΤΜΑ-D11 | Female | 80 | III | Metastasis | 1 | 2 |
| ΤΜΑ-D13 | male | 76 | II | Non-metastasis | 0 | 70 |
| ΤΜΑ-D15 | male | 54 | II | Non-metastasis | 0 | 69 |
| ΤΜΑ-D17 | male | 79 | III | Metastasis | 0 | 69 |
| ΤΜΑ-E01 | Female | 87 | III | Metastasis | 1 | 0.2 |
| ΤΜΑ-E03 | male | 34 | III | Metastasis | 0 | 69 |
| ΤΜΑ-E05 | Female | 68 | II | Non-metastasis | 0 | 69 |
| ΤΜΑ-E07 | Female | 57 | II | Non-metastasis | 0 | 69 |
| ΤΜΑ-E09 | Female | 73 | II | Non-metastasis | 1 | 22 |
| ΤΜΑ-E11 | Female | 83 | II | Non-metastasis | 1 | 23 |
| ΤΜΑ-E13 | male | 84 | II | Non-metastasis | 1 | 8 |
| ΤΜΑ-E15 | Female | 75 | IV | Metastasis | 1 | 58 |
| ΤΜΑ-E17 | male | 76 | III | Metastasis | 0 | 69 |
| ΤΜΑ-F01 | Female | 80 | II | Non-metastasis | 1 | 6 |
| ΤΜΑ-F03 | Female | 81 | III | Metastasis | 1 | 53 |
| ΤΜΑ-F05 | Female | 74 | II | Non-metastasis | 1 | 8 |
| ΤΜΑ-F07 | male | 78 | II | Non-metastasis | 0 | 68 |
| ΤΜΑ-F09 | male | 71 | II | Non-metastasis | 0 | 68 |
| ΤΜΑ-F11 | male | 55 | II | Non-metastasis | 0 | 68 |
| ΤΜΑ-F13 | male | 54 | III | Metastasis | 0 | 68 |
| ΤΜΑ-F15 | male | 57 | III | Metastasis | 1 | 29 |
| ΤΜΑ-F17 | Female | 76 | III | Metastasis | 1 | 56 |
| ΤΜΑ-G01 | Female | 71 | III | Metastasis | 0 | 68 |
| ΤΜΑ-G03 | male | 80 | II | Non-metastasis | 0 | 68 |
| ΤΜΑ-G05 | Female | 53 | III | Metastasis | 1 | 20 |
| ΤΜΑ-G07 | male | 70 | III | Metastasis | 0 | 67 |
| ΤΜΑ-G09 | Female | 84 | II | Non-metastasis | 1 | 39 |
| ΤΜΑ-G11 | Female | 67 | I | Non-metastasis | 0 | 67 |
| ΤΜΑ-G13 | Female | 87 | III | Metastasis | 1 | 5 |
| ΤΜΑ-G15 | Female | 67 | II | Non-metastasis | 0 | 67 |
| ΤΜΑ-G17 | male | 56 | II | Non-metastasis | 0 | 67 |
| ΤΜΑ-H01 | male | 54 | II | Non-metastasis | 0 | 67 |
| ΤΜΑ-H03 | Female | 56 | III | Metastasis | 1 | 57 |
| ΤΜΑ-H05 | male | 74 | III | Metastasis | 0 | 67 |
| ΤΜΑ-H07 | Female | 76 | III | Metastasis | 0 | 67 |
| ΤΜΑ-H09 | Female | 54 | III | Metastasis | 0 | 67 |
| ΤΜΑ-H11 | male | 67 | II | Non-metastasis | 0 | 67 |
| ΤΜΑ-H13 | male | 62 | II | Non-metastasis | 0 | 67 |
| ΤΜΑ-H15 | Female | 61 | III | Metastasis | 1 | 19 |
| ΤΜΑ-H17 | Female | 55 | III | Metastasis | 1 | 29 |
| ΤΜΑ-I01 | male | 73 | II | Non-metastasis | 1 | 32 |
| ΤΜΑ-I03 | male | 63 | II | Non-metastasis | 0 | 66 |
| ΤΜΑ-I05 | Female | 72 | I | Non-metastasis | 0 | 66 |
| ΤΜΑ-I07 | male | 70 | II | Non-metastasis | 1 | 15 |
| ΤΜΑ-I09 | male | 58 | II | Non-metastasis | 0 | 66 |
| ΤΜΑ-I11 | male | 70 | II | Non-metastasis | 0 | 66 |
| ΤΜΑ-I13 | Female | 72 | II | Non-metastasis | 1 | 18 |
| ΤΜΑ-I15 | Female | 77 | II | Non-metastasis | 1 | 17 |
| ΤΜΑ-I17 | male | 67 | II | Non-metastasis | 1 | 7 |
| ΤΜΑ-J01 | male | 49 | II | Non-metastasis | 0 | 66 |
| ΤΜΑ-J03 | male | 61 | II | Non-metastasis | 1 | 29 |
| ΤΜΑ-J05 | male | 81 | II | Non-metastasis | 1 | 24 |
| ΤΜΑ-J07 | male | 60 | II | Non-metastasis | 1 | 68 |
| ΤΜΑ-J09 | male | 65 | II | Non-metastasis | 1 | 13 |
| ΤΜΑ-J11 | Female | 55 | II | Non-metastasis | 0 | 79 |
| ΤΜΑ-J13 | Female | 49 | III | Metastasis | 1 | 14 |
| ΤΜΑ-J15 | male | 69 | II | Non-metastasis | 1 | 43 |
| ΤΜΑ-J17 | Female | 61 | II | Non-metastasis | 0 | 67 |

| **Table S4. Primers used for PCR.** | | | | | |
| --- | --- | --- | --- | --- | --- |
| **Species** | **Gene name** | **Sense** | | **Anti-sense** | **Purpose** |
| **Human** |  | |  |  |  |
|  | *XBP1s* | CCTGGTTGCTGAAGAGGAGG | | CCATGGGGAGATGTTCTGGAG | Splicing Assay |
|  | *XBP1s* | ATGCCCTGGTTGCTGAAGAG | | TGCACCTGCTGCGGACTC | RT-qPCR |
|  | *ACTB* | GCGAGAAGATGACCCAGATC | | CCAGTGGTACGGCCAGAGG | RT-qPCR |
|  | *BIP* | GACGGGCAAAGATGTCAGGA | | GCCCGTTTGGCCTTTTCTAC | RT-qPCR |
|  | *CHOP* | CTGCTTCTCTGGCTTGGCTG | | GCTCTGGGAGGTGCTTGTGA | RT-qPCR |
|  | *VEGFA* | AGGGCAGAATCATCACGAAGT | | AGGGTCTCGATTGGATGGCA | RT-qPCR |
|  | *IL-4* | CCAACTGCTTCCCCCTCTG | | TCTGTTACGGTCAACTCGGTG | RT-qPCR |
|  | *IL-6* | ACTCACCTCTTCAGAACGAATTG | | CCATCTTTGGAAGGTTCAGGTTG | RT-qPCR |
|  | *MMP2* | TACAGGATCATTGGCTACACACC | | GGTCACATCGCTCCAGACT | RT-qPCR |
|  | *PDGFA* | GCAAGACCAGGACGGTCATTT | | GGCACTTGACACTGCTCGT | RT-qPCR |
|  | *ERDJ4* | TCTTAGGTGTGCCAAAATCGG | | TGTCAGGGTGGTACTTCATGG | RT-qPCR |
|  | *THBS1* | CACAGCTCGTAGAACAGGAGG | | CAATGCCACAGTTCCTGATG | RT-qPCR |
|  | *SIRPA* | CAACCAACACGGACTTGTCA | | TGCTCTGCCTGCTGCTC | RT-qPCR |
| **Mouse** |  |  | |  |  |
|  | *Xbp1s* | ACACGCTTGGGAATGGACAC | | CCATGGGAAGATGTTCTGGG | Splicing Assay |
|  | *Xbp1s* | AAGAACACGCTTGGGAATGG | | CTGCACCTGCTGCGGAC | RT-qPCR |
|  | *Actb* | CTCAGGAGGAGCAATGATCTTGAT | | TACCACCATGTACCCAGGCA | RT-qPCR |
|  | *Bip* | ACTTGGGGACCACCTATTCCT | | ATCGCCAATCAGACGCTCC | RT-qPCR |
|  | *Chop* | CTGGAAGCCTGGTATGAGGAT | | CAGGGTCAAGAGTAGTGAAGGT | RT-qPCR |
|  | *Il-4* | GGTCTCAACCCCCAGCTAGT | | GCCGATGATCTCTCTCAAGTGAT | RT-qPCR |
|  | *Il-6* | CTGCAAGAGACTTCCATCCAG | | AGTGGTATAGACAGGTCTGTTGG | RT-qPCR |
|  | *Vegfa* | CCACGACAGAAGGAGAGCAGAAGTCC | | CGTTACAGCAGCCTGCACAGCG | RT-qPCR |
|  | *Mmp2* | CAAGTTCCCCGGCGATGTC | | TTCTGGTCAAGGTCACCTGTC | RT-qPCR |
|  | *Il-33* | TCCAACTCCAAGATTTCCCCG | | CATGCAGTAGACATGGCAGAA | RT-qPCR |
|  | *Pdgfa* | GAGGAAGCCGAGATACCCC | | TGCTGTGGATCTGACTTCGAG | RT-qPCR |
|  | *Tgfb1* | ATGTCACGGTTAGGGGCTC | | GGCTTGCATACTGTGCTGTATAG | RT-qPCR |
|  | *Tnfa* | CCCTCACACTCAGATCATCTTCT | | GCTACGACGTGGGCTACAG | RT-qPCR |
|  | *Erdj4* | TAAAAGCCCTGATGCTGAAGC | | TCCGACTATTGGCATCCGA | RT-qPCR |
|  | *Thbs1* | GTCCACTCAGACCAGGGAGA | | AAAGGTGTCCTGTCCCATCA | RT-qPCR |
|  | *Sirpa* | GGCAACAAGGAGGTCACAGT | | TCCGCGTCCTGTTTCTGTA | RT-qPCR |
|  | *Pd-l1* | AGTATGGCAGCAACGTCACG | | TCCTTTTCCCAGTACACCACTA | RT-qPCR |
|  | *Vegfa* | CCCAAAAGCAGGTCAGTCAC | | CGGTTTGAGGAGGTTGGTTC | ChIP |
|  | *Il-4* | CTGTCCCCTCCCACTACAAA | | GCCTCCTCAGCTCACTCTGT | ChIP |
|  | *Il-6* | GTGTGTGTGTGTGTGTGTGT | | TGGGGCTGATTGGAAACCTT | ChIP |
|  | *Erdj4* | GTAGGGCGCCAAAGTCAG | | TTATAGAGCCGACCTACACGAAA | ChIP |
|  | *Thbs1* | CAAGCGTCTCTGTCATGGAA | | AGCCCTGGAACTTGTCAGAA | ChIP |
|  | *Sirpa* | TTGCCCACTGGAGTCTAAGG | | TGCGCAAACTTGTTTTTCTG | ChIP |
